# Antitumor Effects of Melittin-Functionalized Fe_3_O_4_ Magnetic Nanoparticles in Collagen-I-Based Caco-2/BJ/HUVEC Spheroids Enhanced by Magnetic Hyperthermia

**DOI:** 10.3390/ijms27209302

**Published:** 2026-10-20

**Authors:** Alex Câmpian, Ioana Bâldea, Mara Muntean, Cristian Iacoviță, Nicoleta Decea, Adrian Florea

**Affiliations:** 1 Department of Cell and Molecular Biology, Faculty of Medicine, “Iuliu Hațieganu” University of Medicine and Pharmacy, 6 Louis Pasteur St., 400349 Cluj-Napoca, Romania; alexcampian@yahoo.com (A.C.); muntean.mara@elearn.umfcluj.ro (M.M.); aflorea@umfcluj.ro (A.F.); 2 Department of Physiology, Faculty of Medicine, “Iuliu Hațieganu” University of Medicine and Pharmacy, 1–3 Clinicilor St., 400012 Cluj-Napoca, Romania; nicoleta.decea@yahoo.com; 3 Department of Pharmaceutical Physics-Biophysics, Faculty of Pharmacy, “Iuliu Hațieganu” University of Medicine and Pharmacy, 6 Louis Pasteur St., 400349 Cluj-Napoca, Romania; cristian.iacovita@umfcluj.ro

**Keywords:** melittin-functionalized magnetic nanoparticles, Caco-2 cells, BJ fibroblasts, HUVEC, magnetic hyperthermia, VEGF, HIF-1α, caspase-9, TEM, mitochondria

## Abstract

This study evaluated the integrated biochemical and ultrastructural response of three-dimensional colorectal cancer spheroids to magnetic hyperthermia (MH) mediated by melittin-functionalized Fe_3_O_4_ magnetic nanoparticles (MNPs-Mel). The spheroids comprised human Caco-2 colorectal adenocarcinoma cells, dermal fibroblasts (BJ), and human umbilical vein endothelial cells (HUVECs) in a ratio of 3:1:1 in a collagen-I matrix. They were assigned to control, alternating magnetic field (AMF) control (Control-MH), free melittin (Mel), unloaded MNPs, MNPs-MH, MNPs-Mel, and MNPs-Mel-MH groups and assessed at 24 and 72 h. MTS-reducing activity (colorimetry) and ATP-associated luminescence were quantified as viability markers, while VEGF, HIF-1α, and caspase-9 concentrations measured by ELISA were used to assess angiogenesis and apoptosis induction. MNPs’ size, shape and internalization and treatment-associated ultrastructural changes were examined by transmission electron microscopy (TEM). Treatment, time, and their interaction significantly affected all biochemical endpoints. Every intervention reduced MTS absorbance relative to control at both time points. MNPs-Mel produced lower MTS values than unloaded MNPs, whereas MNPs-Mel-MH generated the lowest ATP signal at both time points and the lowest MTS signal at 72 h. The combined treatment also reduced VEGF and increased caspase-9, maximal at 72 h, while the increase in HIF-1α at 24 h was not maintained at 72 h. TEM showed intracellular MNPs-Mel and a more restricted distribution within spheroids than in the separately examined monocultures. Free Mel and unloaded MNPs produced time-dependent ultrastructural changes, while MH accentuated the changes associated with MNP exposure. MNPs-Mel-MH led to pronounced mitochondrial polymorphism, cristae loss, matrix rarefaction, autophagic-lysosomal alterations, endoplasmic reticulum expansion, reduced intercellular adherence, and extensive disruption of Caco-2 cells, particularly at 72 h. However, biochemical endpoints represent the response of the whole spheroid and do not distinguish effects on Caco-2 cells from those on BJ fibroblasts or HUVECs. The molecular and ultrastructural findings identify MNPs-Mel-MH as the treatment associated with the broadest response in this tri-cellular spheroid model, linking sustained metabolic suppression and reduced pro-angiogenic signaling with marked mitochondrial and cellular injury.

## 1. Introduction

Cancer remains a major cause of morbidity and mortality worldwide. Estimates for 2024 indicated approximately 20.6 million new cancer cases and 9.8 million cancer-related deaths, with colorectal cancer accounting for about 9.9% of new diagnoses and 9.4% of cancer deaths globally [1]. Colorectal cancer development and treatment response are influenced not only by malignant epithelial cells but also by the tumor microenvironment, which contains fibroblasts, endothelial cells, extracellular-matrix components and soluble signaling molecules [2,3]. For in vitro studies, conventional two-dimensional cultures reproduce only a limited part of this biological complexity because they do not adequately generate the cell–cell interactions, extracellular-matrix contacts and oxygen or nutrient gradients found in solid tumors. Multicellular spheroids provide a more physiologically relevant intermediate between monolayer cultures and animal models, as increasing the spheroid size can produce spatial differences in proliferation, differentiation, nutrient availability and hypoxia-associated signaling [2]. Hypoxia and glucose deprivation in multicellular spheroids have been shown to stimulate vascular endothelial growth factor (VEGF) expression, while extracellular-matrix composition can modify epithelial polarity, transporter expression and drug sensitivity [2,4]. Nevertheless, spheroids should not be regarded as complete replicas of tumors, since they reproduce only selected components of the tumor microenvironment. In the present study, a collagen-I-based tricellular spheroid model containing Caco-2 colorectal adenocarcinoma cells, BJ fibroblasts and human umbilical vein endothelial cells was therefore used to incorporate malignant, stromal and endothelial components within a controlled three-dimensional system. The complex structures are simply called spheroids throughout the paper.

The Caco-2 cell line was established from human colon adenocarcinoma and grows as an adherent epithelial population [5]. When maintained beyond confluence under appropriate culture conditions, these cells can develop apical–basal polarity, intercellular junctions, microvilli and brush-border enzyme activity, producing an enterocyte-like phenotype [5,6]. These characteristics have made Caco-2 cells a widely used model of intestinal epithelial differentiation and barrier function, as well as a model for evaluating the cellular effects of experimental antitumor treatments [5,6,7]. Their ability to form organized epithelial structures is particularly relevant in three-dimensional models, in which extracellular-matrix contact, and spatial organization may influence treatment susceptibility [2]. However, the differentiated phenotype of Caco-2 cells also means that they do not represent the full molecular and phenotypic heterogeneity of colorectal cancer. Results obtained with this line should therefore be interpreted as responses of a defined colorectal adenocarcinoma model rather than as effects applicable to every colorectal tumor [5,6,7]. In a previous study relevant to the present work, Caco-2 cells were exposed to citrate-coated Fe_3_O_4_ magnetic nanoparticles bearing surface-adsorbed melittin (Mel). The combined nanoparticle–melittin formulation reduced metabolic viability and cellular ATP, increased lactate dehydrogenase release and caspase-9 levels, and produced ultrastructural alterations; several of these effects were enhanced by magnetic hyperthermia (MH) [7]. Extending this approach to a multicellular spheroid may provide a more stringent assessment of treatment activity in the presence of extracellular matrix and non-malignant cellular components.

Fibroblasts are important stromal cells that synthesize, organize and remodel extracellular-matrix components and can influence tumor-cell behavior through direct contacts, matrix-mediated signals and secreted factors [3]. In established tumors, fibroblasts can acquire heterogeneous cancer-associated phenotypes that support matrix deposition, angiogenesis and tumor progression [3]. BJ cells, however, are normal human dermal fibroblasts derived from neonatal foreskin and should not automatically be described as cancer-associated fibroblasts. They are telomerase-negative, have a finite replicative lifespan and are commonly used as a non-transformed fibroblast model [8]. Their incorporation into the present spheroid provides both a stromal component and a normal-cell comparator for assessing nonspecific toxicity. Including BJ cells therefore allows treatment activity to be examined in the presence of a non-tumoral stromal population that can influence the overall spheroid response. Studies using three-dimensional collagen models have demonstrated that BJ fibroblasts can contribute to fibrillar collagen organization and that their position relative to tumor cells can influence tumor-cell invasion, illustrating the importance of spatial organization in multicellular cultures [9]. Nevertheless, their dermal origin and absence of experimentally demonstrated cancer-associated activation must be recognized as model limitations. In the previous Caco-2/BJ study, free melittin affected both cell types, although Caco-2 cells generally displayed greater sensitivity at lower concentrations and a stronger delayed response to the nanoparticle–melittin–hyperthermia combination [7]. Including BJ cells in the present spheroid is consequently important for determining whether treatment responses remain preferentially associated with malignant cells or also affect the surrounding normal stromal population.

Endothelial cells constitute another essential component of the tumor microenvironment because tumor expansion depends on the exchange of oxygen, nutrients and metabolic products and is frequently accompanied by angiogenic signaling. HUVECs are primary endothelial cells isolated from human umbilical veins and retain characteristic endothelial features, including a polygonal or cobblestone-like morphology and Weibel–Palade bodies [10]. They are frequently used to investigate endothelial survival, migration, organization and responses to angiogenic mediators. In three-dimensional tumor–endothelial co-cultures, tumor-derived vascular endothelial growth factor (VEGF) can promote HUVEC sprouting and lumen-like organization, whereas hypoxia can enhance these responses and VEGF inhibition can suppress them [11]. Hypoxia-associated angiogenic signaling is especially relevant in spheroids because the diffusion of oxygen and nutrients becomes increasingly restricted toward the center of sufficiently large aggregates [4]. At the molecular level, hypoxia-inducible factor 1 (HIF-1) stimulates VEGF transcription through a hypoxia-responsive regulatory element in the VEGF gene [12]. Consequently, measurements of HIF-1α and VEGF can provide information about hypoxia- and angiogenesis-associated signaling in the tricellular model. They do not, by themselves, demonstrate the formation of functional blood vessels. Similarly, the inclusion of HUVECs provides an endothelial component but should not be described as producing a perfused or fully vascularized spheroid unless interconnected lumens and functional perfusion are experimentally demonstrated [10,11,12].

Magnetic iron-oxide nanoparticles (MNPs) are being investigated as platforms for externally controlled hyperthermia and drug or bioactive-molecule delivery. Magnetite nanoparticles can exhibit superparamagnetic behavior when their magnetic domains are sufficiently small, allowing them to respond strongly to an applied magnetic field while showing minimal residual magnetization after the field is removed [13]. Under an alternating magnetic field, magnetic energy can be converted into heat through relaxation-associated processes, including Néel and Brownian relaxation, although the relative contribution of each mechanism depends on the particle size, magnetic anisotropy, aggregation, viscosity and surface immobilization [13]. Heating efficiency also depends on the nanoparticle concentration and the amplitude and frequency of the alternating magnetic field; therefore, hyperthermia results obtained with one formulation or field configuration cannot automatically be generalized to another [13]. The Fe_3_O_4_ particles characterized in the preceding study had an average diameter of approximately 45 nm, an inverse-spinel magnetite structure and superparamagnetic behavior at room temperature. Melittin was adsorbed onto the negatively charged citrate-coated surface of MNPs, producing a melittin-functionalized MNP (MNPs-Mel) formulation [7]. MNPs may also facilitate local concentration of a surface-associated therapeutic agent, while field-induced heating can independently damage susceptible cellular structures or increase treatment activity. Magnetic hyperthermia has reduced tumor-cell viability in three-dimensional spheroid models, supporting the evaluation of such systems under conditions that better represent the diffusion barriers of solid tumors [14]. At the same time, the extracellular matrix, spheroid size, nanoparticle aggregation and cellular uptake can affect both the particle distribution and heat transfer. Direct assessment in a tricellular collagen spheroid is therefore necessary before conclusions drawn from monolayer cultures can be extended to a more complex tumor model.

Melittin is a 26-amino-acid, cationic, amphipathic peptide and a major membrane-active component of honeybee venom [15]. Its interaction with phospholipid membranes can produce transient membrane permeabilization and, at higher or sustained exposure, membrane disruption and loss of cellular integrity [15]. Beyond direct membrane effects, Mel has demonstrated anticancer activity through several intracellular mechanisms. In colorectal cancer cell models, Mel increased mitochondrial reactive oxygen species, reduced mitochondrial membrane potential, altered the Bax/Bcl-2 balance, promoted cytochrome-c release and increased caspase-3, -7 and -9 activity, supporting involvement of the intrinsic mitochondrial apoptotic pathway [16]. Additional studies in colorectal cancer cells have linked Mel-induced apoptosis to disruption of calcium homeostasis and endoplasmic-reticulum stress [17]. A colorectal-cancer-focused study also found that direct Mel exposure inhibited HUVEC tube formation and reduced VEGF secretion in HUVEC cultures, suggesting that endothelial responses may contribute to its overall biological activity [16]. However, the membrane activity responsible for Mel’s antitumor effects can also damage normal cells and erythrocytes, limiting systemic administration. Nanoparticle-based formulations have consequently been explored to improve delivery and reduce nonspecific toxicity; for example, incorporation of Mel into a zeolitic imidazolate framework reduced hemolysis while preserving antitumor activity in experimental models [18]. Surface adsorption of Mel onto Fe_3_O_4_ nanoparticles provides another delivery strategy and can be combined with alternating magnetic field (AMF) exposure [7].

The aim of the present study was therefore to investigate the effects of Mel-functionalized Fe_3_O_4_ nanoparticles, with and without MH, in collagen-I-based Caco-2/BJ/HUVEC spheroids. Metabolic viability, cellular ATP status, VEGF/HIF-1α-associated responses, caspase-9-related apoptotic signaling and ultrastructural alterations were examined to characterize treatment activity across malignant, stromal and endothelial components.

## 2. Results

### 2.1. Melittin-Functionalized Magnetic Nanoparticles

Melittin-functionalized nanoparticles were successfully prepared using the previously reported adsorption procedure [7]. The MNPs exhibited a spherical morphology and had an average diameter of approximately 45 nm (Figure S1) while UV–Vis analysis of the unbound peptide fraction indicated an apparent loading of 54.57 µg Mel per mg of MNPs [7]. These findings support preparation of the MNPs-Mel formulation used in the present spheroid experiments. Structural analysis confirmed a pure inverse spinel crystalline phase, consistent with magnetite (Fe_3_O_4_). These MNPs demonstrated a saturation magnetization of 80 emu/g and a specific absorption rate ranging from 110 to 2900 W/g under magnetic field amplitudes of 10–60 kA/m, as previously reported [7].

### 2.2. Magnetic Hyperthermia and Melittin Release

During AMF exposure, the measured surface temperatures of the spheroid preparations approached plateaus of approximately 38.1 °C for MNPs and 38.6 °C for MNPs-Mel, compared with 37.6 °C in the control condition (Figure S2). These measurements documented the thermal response under the conditions used for the biological experiments.

For the Mel release assay, the presence of adsorbed Mel is demonstrated in Figure S3, where the black line shows the initial concentration of Mel in the used solution, and the red line shows the concentration of Mel in the supernatant, after the removal of MNPs-Mel. During the MH exposure, the MNPs-Mel samples reached a temperature of approximately 43 °C after 7 min, which was preserved near this value for a further 3 min (Figure S4). Following magnetic separation of the MNPs, the recovered supernatants exhibited absorbance in the Mel-associated spectral region, consistent with the presence of soluble Mel (Figure S5). Together, the heating measurements and the detection of soluble Mel provide experimental context for considering the potential peptide availability when interpreting the biological effects of MNPs-Mel-MH.

### 2.3. MTS Assay

**Figure 1:**
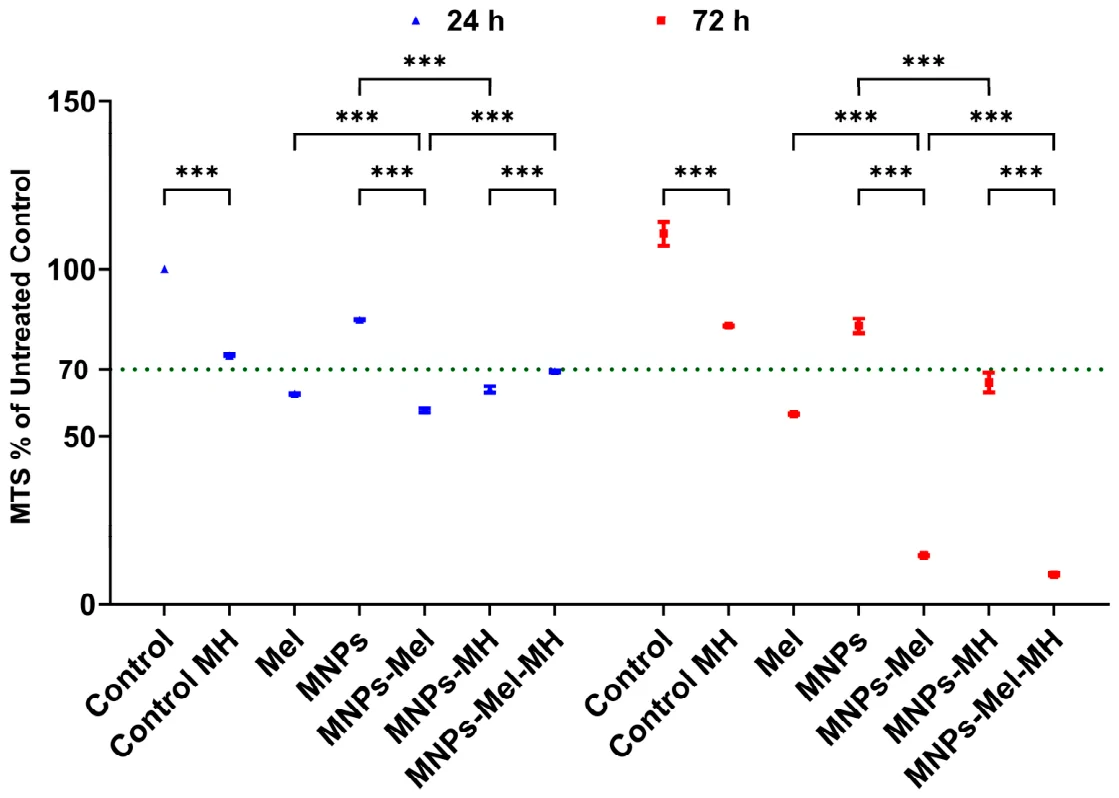
Overall metabolic viability in collagen-I-based Caco-2/BJ/HUVEC spheroids exposed to Mel (2.5 µg/mL), MNPs and MNPs-Mel (50 µg/mL), with and without magnetic hyperthermia, at two time points (24 h and 72 h). Data are presented as mean ± SD (*n* = 4). *** = *p* < 0.001.

MTS results are expressed as percentages of the untreated 24 h control within each independent experiment (mean ± SD; *n* = 4 independent experiments). The same reference was used for all groups at both intervals, including the untreated 72 h control. The 24 h (blue triangles in Figure 1) and 72 h (red squares in Figure 1) labels indicate the initial incubation intervals; all groups underwent the same additional 24 h recovery period before measurement. The 24 h control was therefore 100% by normalization.

Ordinary two-way ANOVA identified significant effects of the treatment [F(6, 42) = 2415.04], incubation interval [F(1, 42) = 1155.12], and their interaction [F(6, 42) = 756.20] (all *p* < 0.001). Pairwise comparisons used Tukey’s multiple comparisons test across all 21 treatment comparisons within each interval. All pairwise *p*-values reported below are adjusted.

At 24 h, all five active treatment groups had lower MTS responses than the untreated control (all *p* < 0.001). MNPs-Mel produced the lowest mean response (57.75 ± 0.65%), which was numerically lower than free Mel (62.65 ± 0.17%), although this difference was not statistically significant (*p* = 0.8034). MNPs-Mel was lower than MNPs-Mel-MH (69.38 ± 0.29%; *p* < 0.001). Control-MH, which received field exposure without Mel or nanoparticles under otherwise matched conditions, also showed a reduced response (74.18 ± 0.17% vs. 100%; *p* < 0.001).

At 72 h, MNPs-Mel-MH produced the lowest MTS response (8.80 ± 0.27%), followed by MNPs-Mel (14.55 ± 0.06%; *p* < 0.001 for this comparison). All five active treatment groups remained below the corresponding untreated control (110.40 ± 3.58%; all *p* < 0.001). Control-MH was also lower than the untreated control (83.00 ± 0.12%; *p* < 0.001). MNPs-Mel-MH remained below Control-MH at 24 h (*p* = 0.0004) and 72 h (*p* < 0.001). MNPs-MH yielded lower responses than MNPs alone at 24 h (64.05% vs. 84.93%; *p* < 0.001) and 72 h (66.08% vs. 82.98%; *p* < 0.001). Thus, the effect of AMF exposure on MNPs-Mel-treated spheroids depended on the incubation interval: the combined treatment retained a higher MTS signal at 24 h but produced the greatest MTS suppression at 72 h.

### 2.4. ATP Assay

ATP results were expressed using the same 24 h control reference and analyzed using the same statistical procedures as the MTS data (mean ± SD; *n* = 4 independent experiments). Ordinary two-way ANOVA identified significant effects of treatment [F(6, 42) = 1068.11], incubation interval [F(1, 42) = 3644.56], and their interaction [F(6, 42) = 154.57] (all *p* < 0.001). Pairwise comparisons used Tukey’s multiple comparisons test within each interval.

**Figure 2:**
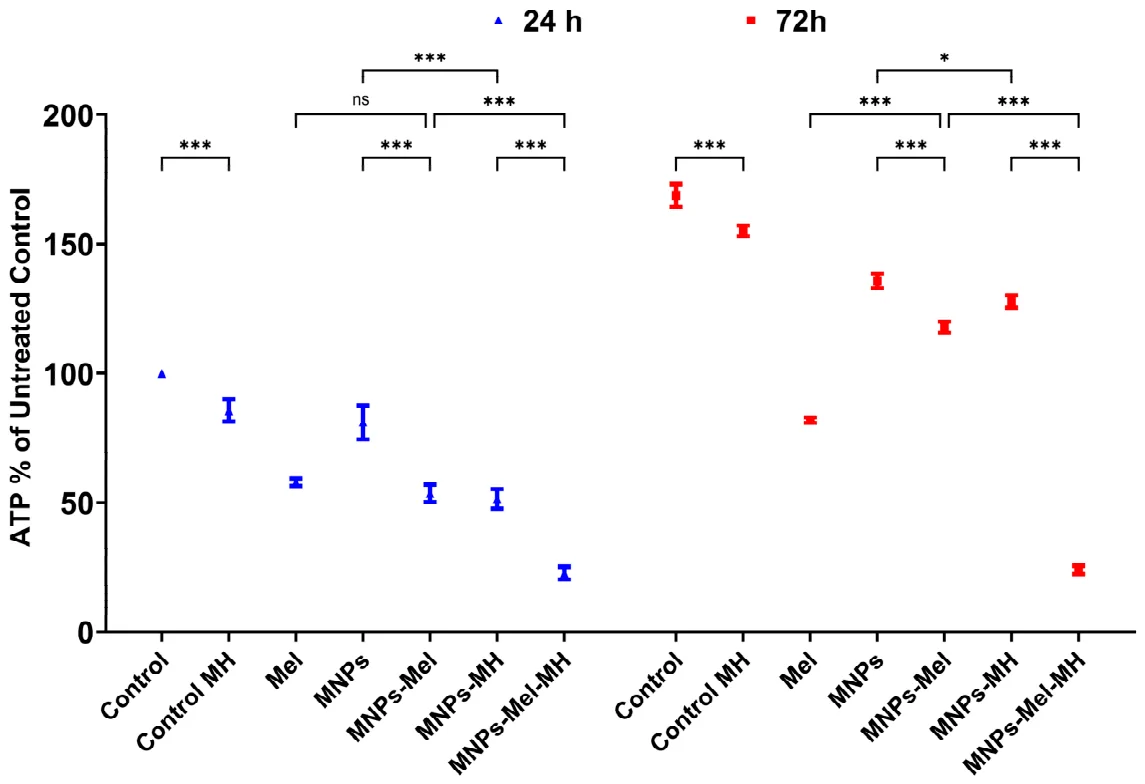
Overall ATP measurements in collagen-I-based Caco-2/BJ/HUVEC spheroids exposed to Mel (2.5 µg/mL), MNPs and MNPs-Mel (50 µg/mL), with and without magnetic hyperthermia, at two time points (24 h and 72 h). Data are presented as mean ± SD (*n* = 4). * = *p* < 0.05, *** = *p* < 0.001, ns = not significant.

At 24 h (blue triangles in Figure 2), all five active treatment groups had lower ATP signals than untreated control (all adjusted *p* < 0.001). MNPs-Mel-MH produced the lowest mean signal (22.78 ± 2.45%), which was lower than both MNPs-Mel (53.55 ± 3.44%) and free Mel (57.90 ± 1.52%; both *p* < 0.001). MNPs-Mel yielded a numerically lower signal than free Mel, but this difference was not statistically significant (*p* = 0.0783). Control-MH was lower than untreated control (85.63 ± 4.29% vs. 100%; *p* < 0.001), and MNPs-Mel-MH remained below Control-MH (*p* < 0.001).

At 72 h (red squares in Figure 2), MNPs-Mel-MH again produced the lowest ATP signal (24.08 ± 1.55%), which remained below MNPs-Mel and free Mel (both *p* < 0.001). MNPs-Mel yielded a higher signal than free Mel (117.83 ± 2.35% vs. 82.00 ± 1.11%; *p* < 0.001), but remained below the corresponding untreated control (168.80 ± 4.38%; *p* < 0.001). MNPs-MH was lower than MNPs alone (127.68% vs. 135.60%; *p* < 0.001). Control-MH was also lower than untreated control (155.00 ± 2.25% vs. 168.80 ± 4.38%; *p* < 0.001), whereas MNPs-Mel-MH remained below Control-MH (*p* < 0.001). ATP values above 100% at 72 h therefore still represented reduced signals relative to the untreated control at the same interval. The combined treatment produced the most pronounced ATP reduction at both intervals, indicating a consistent effect on spheroid ATP content despite the different temporal pattern observed with MTS.

### 2.5. Vascular Endothelial Growth Factor

Supernatant VEGF concentration was affected by the experimental condition [F(6, 28) = 244.22], initial incubation interval [F(1, 28) = 264.99] and their interaction [F(6, 28) = 54.00] (all *p* < 0.001). Tukey-adjusted pairwise comparisons were performed among the seven conditions separately at each interval.

**Figure 3:**
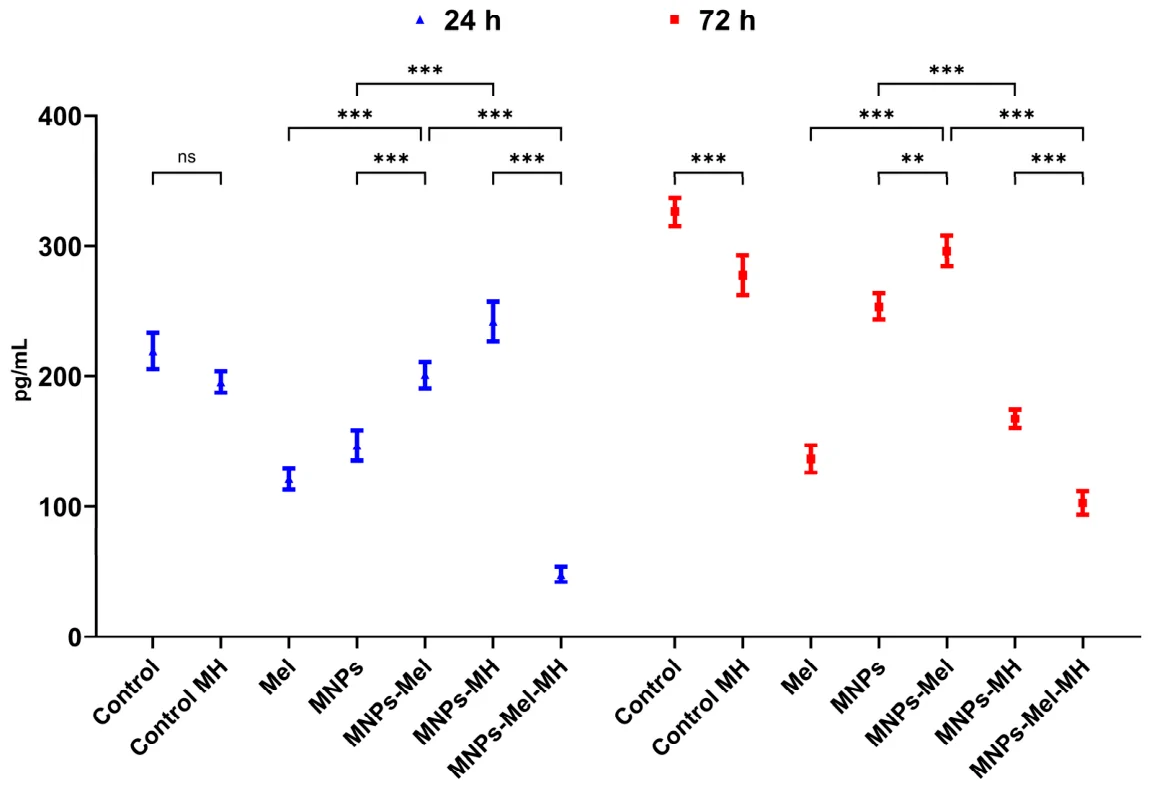
VEGF concentrations in collagen-I-based Caco-2/BJ/HUVEC spheroids exposed to Mel (2.5 µg/mL), MNPs and MNPs-Mel (50 µg/mL), with and without magnetic hyperthermia, at two time points (24 h and 72 h). Data are presented as mean ± SD (*n* = 3). ** = *p* < 0.01, *** = *p* < 0.001, ns = not significant.

At 24 h (blue triangles in Figure 3), VEGF was lower than the untreated control after Mel, MNPs and MNPs-Mel-MH exposure (all adjusted *p* < 0.001). Control-MH (*p* = 0.1432), MNPs-Mel (*p* = 0.4010) and MNPs-MH (*p* = 0.1974) did not differ significantly from the control. MNPs-MH produced a higher VEGF than MNPs alone, and MNPs-Mel produced a higher VEGF than Mel or MNPs (all *p* < 0.001). MNPs-Mel-MH had the lowest mean concentration and was lower than every other condition (all *p* < 0.001).

At 72 h (red squares in Figure 3), VEGF was lower than the control in all five treatment groups and in Control-MH. The MNPs-Mel comparison had an adjusted *p* = 0.0309; the other comparisons with Control had *p* < 0.001. MNPs-MH was lower than MNPs alone (*p* < 0.001). MNPs-Mel remained higher than Mel (*p* < 0.001) and MNPs (*p* = 0.001005). MNPs-Mel-MH had the lowest mean concentration, with lower values than Mel (*p* = 0.0119) and all remaining conditions (*p* < 0.001).

### 2.6. HIF-1α

**Figure 4:**
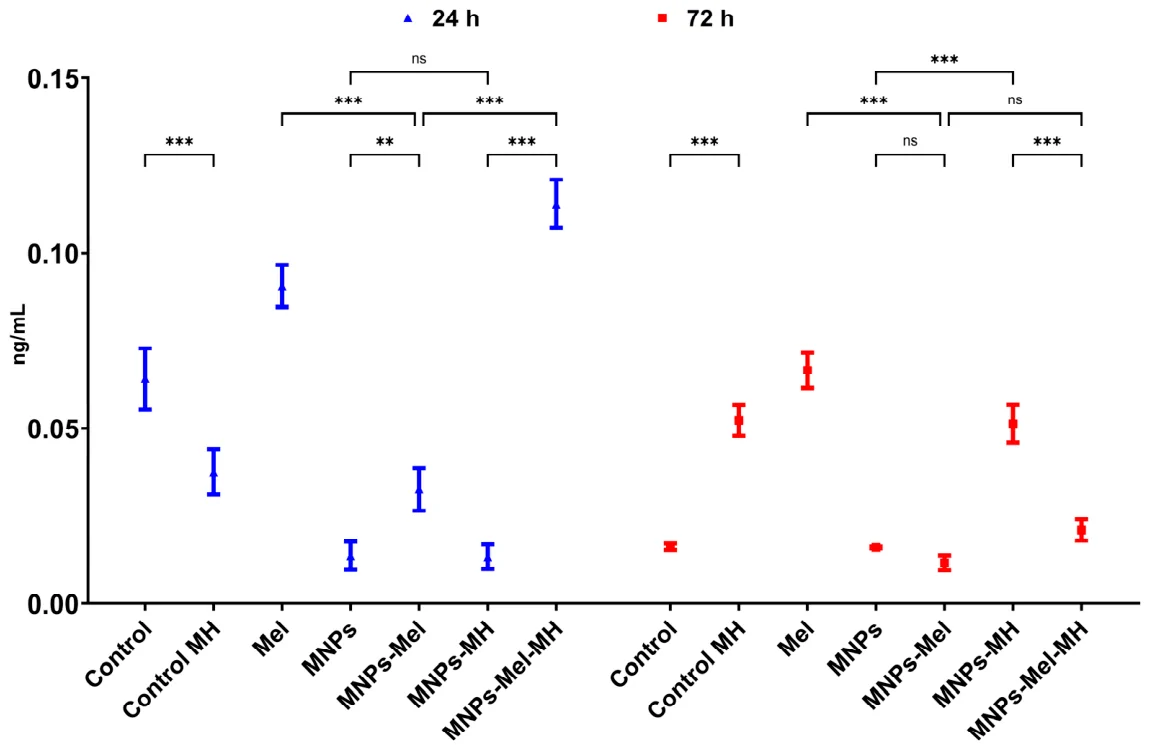
HIF-1α concentrations in collagen-I-based Caco-2/BJ/HUVEC spheroids exposed to Mel (2.5 µg/mL), MNPs and MNPs-Mel (50 µg/mL), with and without magnetic hyperthermia, at two time points (24 h and 72 h). Data are presented as mean ± SD (*n* = 3). ** = *p* < 0.01, *** = *p* < 0.001, ns = not significant.

The supernatant HIF-1α concentration was affected by the experimental condition [F(6, 28) = 127.03], initial incubation interval [F(1, 28) = 145.11] and their interaction [F(6, 28) = 110.15] (all *p* < 0.001). At 24 h (blue triangles in Figure 4), Mel and MNPs-Mel-MH produced higher concentrations than the control, whereas Control-MH, MNPs, MNPs-Mel and MNPs-MH produced lower concentrations (all adjusted *p* < 0.001). MNPs and MNPs-MH did not differ significantly (*p* > 0.9999). MNPs-Mel was lower than Mel (*p* < 0.001) and higher than MNPs (*p* = 0.0014). MNPs-Mel-MH was higher than MNPs-Mel and Control-MH (both *p* < 0.001).

At 72 h (red squares in Figure 4), Control-MH, Mel and MNPs-MH had higher HIF-1α concentrations than Control (all *p* < 0.001). MNPs (*p* > 0.9999), MNPs-Mel (*p* = 0.9224) and MNPs-Mel-MH (*p* = 0.8979) did not differ significantly from control. MNPs-MH was higher than MNPs (*p* < 0.001), but did not differ from control-MH (*p* > 0.9999). MNPs-Mel was lower than Mel (*p* < 0.001), but did not differ significantly from MNPs (*p* = 0.9387) or MNPs-Mel-MH (*p* = 0.2903). MNPs-Mel-MH was lower than Control-MH (*p* < 0.001). The elevation relative to Control observed with MNPs-Mel-MH at 24 h was absent at 72 h. The HIF-1α increase associated with MNPs-Mel-MH at 24 h was not maintained at 72 h, demonstrating a temporal pattern distinct from the sustained reduction in VEGF.

### 2.7. Caspase-9

**Figure 5:**
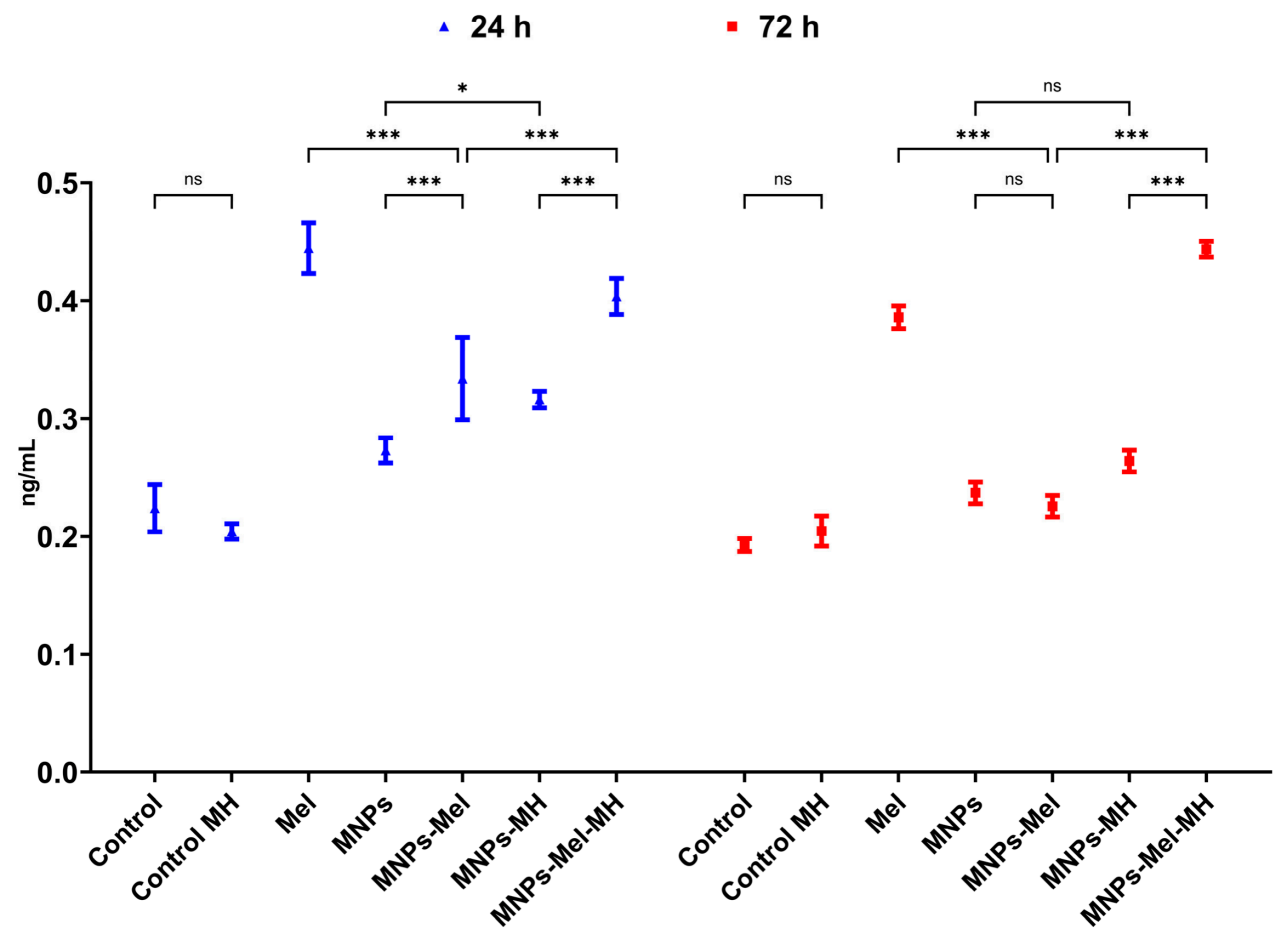
Caspase-9 concentrations in collagen-I-based Caco-2/BJ/HUVEC spheroids exposed to Mel (2.5 µg/mL), MNPs and MNPs-Mel (50 µg/mL), with and without magnetic hyperthermia, at two timepoints (24 h and 72 h). Data are presented as mean ± SD (*n* = 3). * = *p* < 0.05, *** = *p* < 0.001, ns = not significant.

The immunoreactive caspase-9 concentration in culture supernatant was affected by the experimental condition [F(6, 28) = 218.53], initial incubation interval [F(1, 28) = 58.50] and their interaction [F(6, 28) = 14.76] (all *p* < 0.001). At 24 h (blue triangles in Figure 5), all five treatment groups had higher concentrations than the control: MNPs, adjusted *p* = 0.0064; all other treatments, *p* < 0.001. Control-MH did not differ significantly from the control (*p* = 0.6738). Free Mel produced the highest mean concentration and was higher than MNPs-Mel-MH (*p* = 0.0319). MNPs-MH was higher than MNPs (*p* = 0.0203). MNPs-Mel was lower than Mel and higher than MNPs (both *p* < 0.001); MNPs-Mel-MH was higher than MNPs-Mel and Control-MH (both *p* < 0.001).

At 72 h (red squares in Figure 5), caspase-9 concentrations were higher than the control after Mel, MNPs-MH and MNPs-Mel-MH exposure (all *p* < 0.001), and after MNPs exposure (*p* = 0.0166). Control-MH (*p* = 0.9530) and MNPs-Mel (*p* = 0.1326) did not differ significantly from the control. MNPs and MNPs-MH did not differ significantly (*p* = 0.3181), and MNPs-Mel did not differ from MNPs (*p* = 0.9641). MNPs-Mel alone showed a weaker caspase-9 response at the later interval, whereas AMF exposure of MNPs-Mel-treated spheroids maintained an elevated concentration and produced the highest value at 72 h. These measurements describe extracellular protein concentration and do not directly demonstrate caspase-9 activation.

### 2.8. Transmission Electron Microscopy Evaluation

#### 2.8.1. General Structure of Spheroids

**Figure 6:**
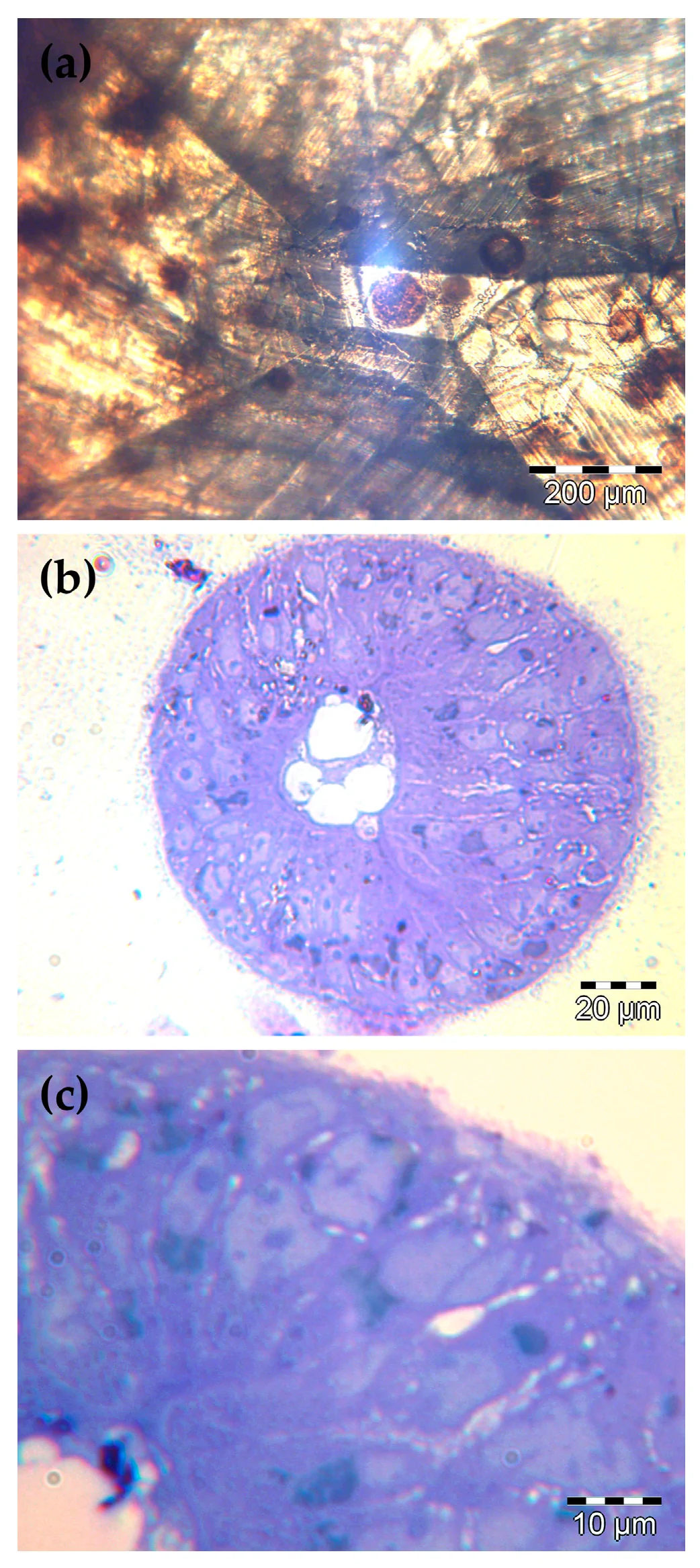
Representative light microscopy images with a spheroid in control group: (**a**) upper view of polymerized EMBed-812 block after manual trimming at the level of a spheroid. (**b**) Low magnification (obj. ×40) that allowed observation of an entire spheroid on a semithin section. (**c**) High magnification (obj. ×100) of the upper right part of the same spheroid.

The spheroid samples processed for TEM were preliminary used to assess their morphology. Figure 6a presents the tip of a polymerized epoxy resin block with spheroids from the control group, manually trimmed, and before cutting the semithin sections. Examination of semithin sections confirmed the actual spherical structure of the cultivated spheroids (Figure 6b,c). The Caco-2 cells were distributed at the periphery of the spheroids and showed numerous microvilli at the apical pole, while the BJ cells and the HUVECs occupied the internal region of the structure. Cell death occurred in the central region of the spheroid, resulting in several large empty vacuoles.

#### 2.8.2. Internalization Control

**Figure 7:**
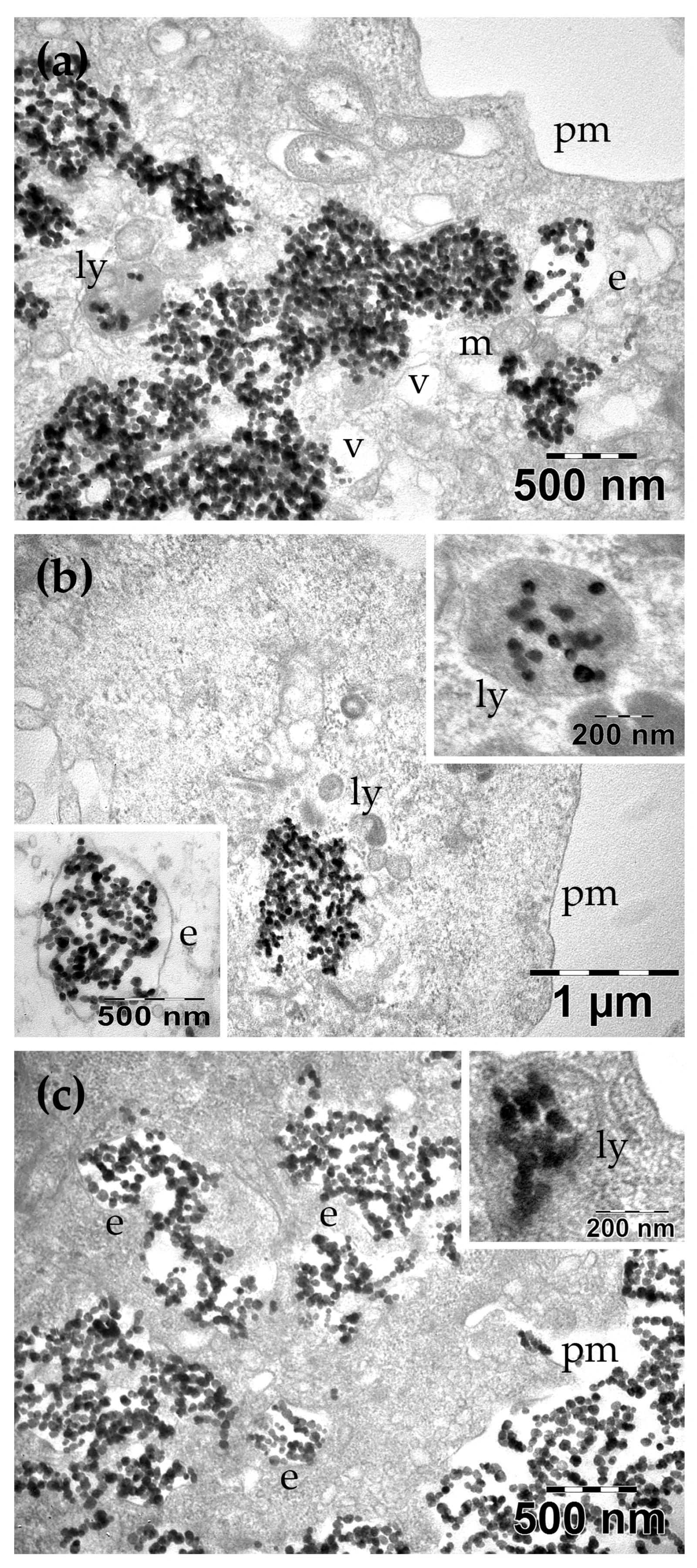
Representative TEM images demonstrating cellular internalization of nanoparticles after 4 h incubation with MNPs-Mel in BJ (**a**), HUVEC (**b**) and Caco-2 (**c**) cells. e: endosome; ly: lysosome; pm: plasma membrane; v: vesicle.

The 4 h incubation with MNPs-Mel showed the ability of all three types of cells to internalize nanoparticles in high amounts. Thus, the MNPs-Mel were found inside endosomes, in lysosomes and dispersed free in the cytosol in BJ cells (Figure 7a), HUVECs (Figure 7b) and Caco-2 cells (Figure 7c).

#### 2.8.3. Control Group

**Figure 8:**
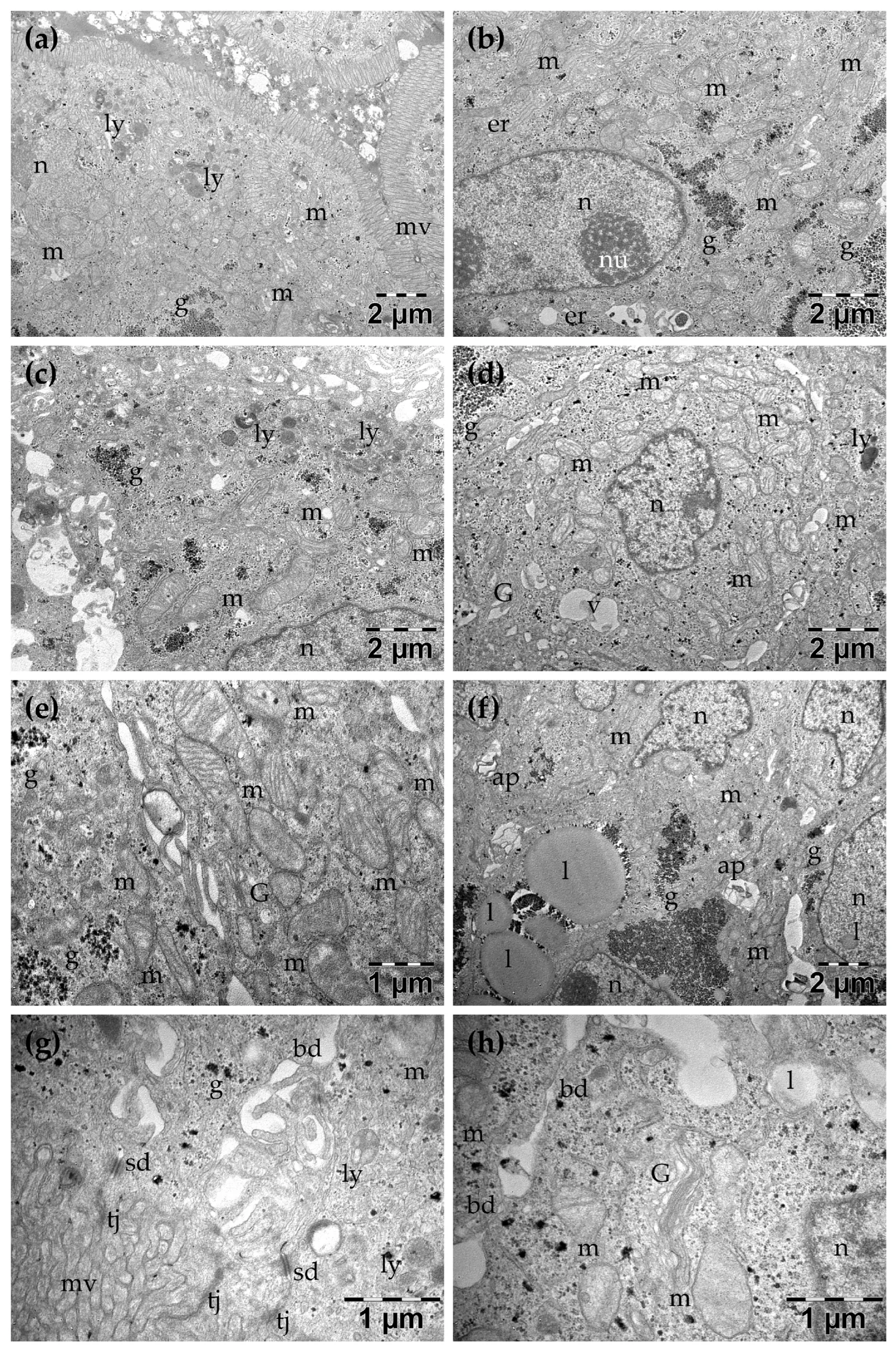
(**a**–**h**) Representative TEM images of cells in spheroids from control group. ap: autophagosome; bd: belt desmosome; er: endoplasmic reticulum; G: Golgi apparatus; g: glycogen; l: lipid droplets; ly: lysosome; m: mitochondria; mv: microvilli; n: nucleus; sd: spot desmosomes; tj: tight junction; v: vesicle.

The outer layer(s) of the spheroids consisted of Caco-2 cells, and their interior was formed by BJ cells and HUVECs as well as by rare undifferentiated Caco-2 cells that could not be identified on the TEM images. While in monoculture, the Caco-2 cells had fewer thin extensions of plasma membrane ; once organized in spheroids, they displayed a continuous border of microvilli (Figure 8a). These cells had large, euchromatic nuclei (Figure 8b), and the cytoplasm containing numerous organelles: mitochondria of round/oval shape, rare profiles of endoplasmic reticulum, and primary and secondary lysosomes (Figure 8a–c). Also, many glycogen granules were dispersed within their cytoplasm (Figure 8a–c). The BJ cells and HUVECs were difficult to identify distinctly deeper inside the spheroids.

They had more elongated mitochondria, some vacuoles and rare granules of glycogen (Figure 8d,e). Some Caco-2 cells contained lipid droplets of 1–4 µm and with homogeneous content, and rare autophagosomes (Figure 8f). Furthermore, lipid droplets of small size and homogeneous aspect were also noted inside the nuclei of these cells (Figure 8f). The Caco-2 cells specifically interacted with each other by tight junctions and desmosomes (spot and belt desmosomes), while the contacts with the other cells were established by belt desmosomes (Figure 8g,h).

#### 2.8.4. Control-MH 24 h Group and Control-MH 72 h Group

The general architecture of the spheroids was preserved consecutive to the 24 h (Figure S6a,b) and 72 h (Figure S6c,d) exposure to the AMF. Endoplasmic reticulum had a normal ultrastructure in all cell types, autophagosomes were rare and glycogen granules were found in normal amounts. Mitochondria preserved their round-oval shapes, but at 72 h, rare mitochondria had increased size (Figure S6c). At both timepoints, mitochondria with a reduced number of cristae were present. Moreover, in both groups exposed to AMF, the lipid droplets from the Caco-2 cells had different electron densities, some of them being impossible to distinguish from primary lysosomes. Additionally, a heterogeneity of the lipid droplets was noted (Figure S6b,c), many of them containing a peripheral electron-dense region with multiple round-oval myelin-like figures.

#### 2.8.5. Mel 24 h Group

The spheroids incubated for 24 h with Mel showed moderate ultrastructural changes concerning mainly the Caco-2 cells. They reacted to the Mel treatment by reduction (in number and size) of their microvilli (Figure S7a,b,d,e) and by increasing their lipid content (Figure S7a,b). Also, many of their mitochondria were either ballooned (Figure S7a) or elongated (Figure S7b), with fewer cristae and electron-lucent matrix. On the other hand, some cells showed only rare polymorphous mitochondria (ring or V-shaped), or mitochondria almost completely devoid of cristae (Figure S7c,d), while the other organelles had a normal ultrastructure. A particular aspect noted in the Caco-2 cells was the presence of lipid droplets with a heterogeneous aspect (Figure S7a,e,f), some of them having a peripheral denser region (Figure S7e,f), an aspect similar to that reported for the Control-MH groups.

#### 2.8.6. Mel 72 h Group

**Figure 9:**
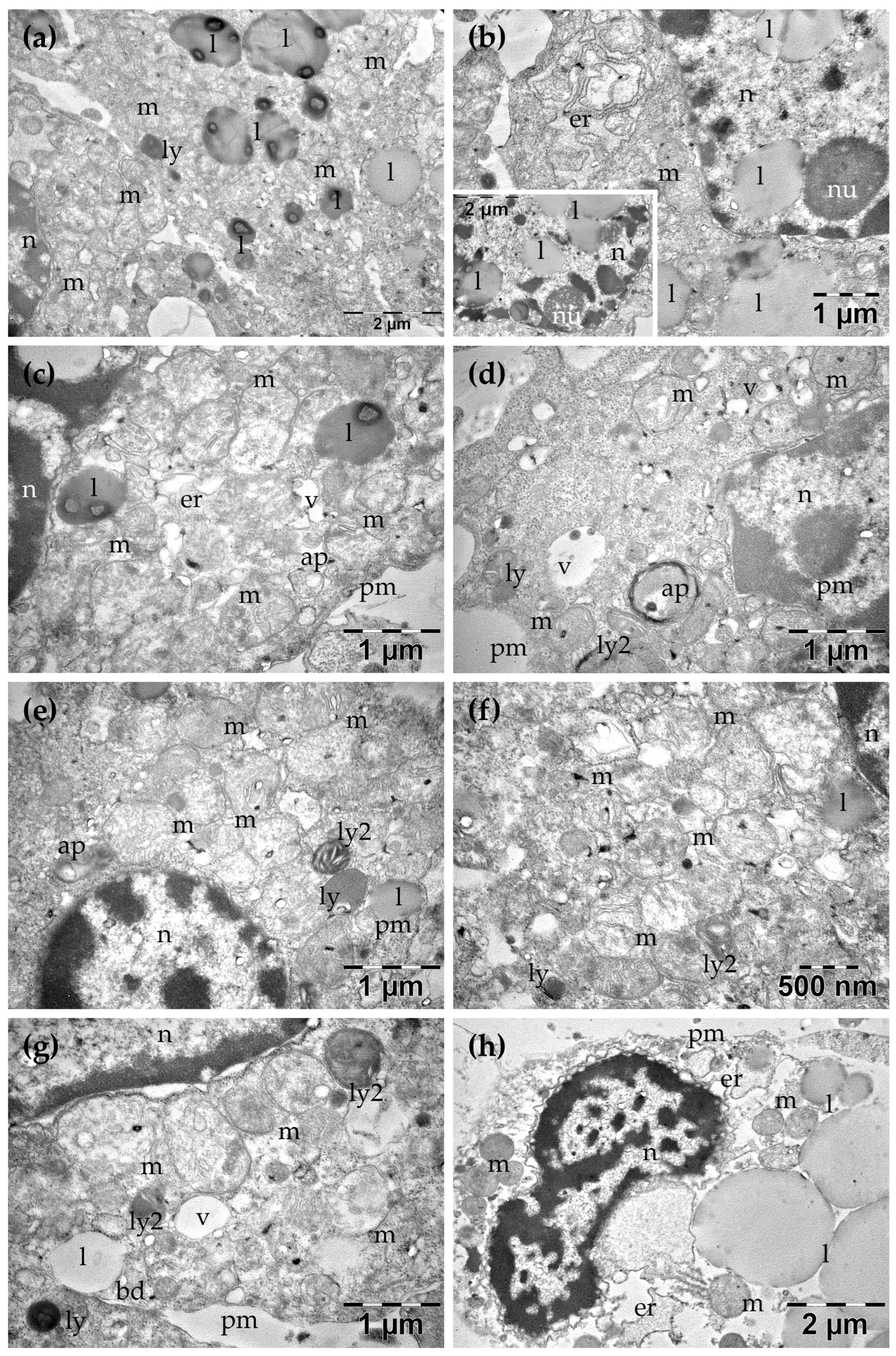
(**a**–**h**) Representative TEM images of cells in spheroids from Mel 72 h group. ap: autophagosome; bd: belt desmosome; er: endoplasmic reticulum; l: lipid droplets; ly: lysosome; ly2: secondary lysosome; m: mitochondria; n: nucleus; nu: nucleolus; pm: plasma membrane; v: vesicle.

The spheroids preserved generally unchanged architecture consecutively to the 72 h incubation with Mel, but the cells showed a tendency to diminish their connections (Figure 9a,b). However, the number of lipid droplets was much increased in the Caco-2 cells as compared to the 24 h incubation, most of them with a heterogeneous aspect (Figure 9a–c). Moreover, an increased content of nuclear lipids was noted in the cells of this group (Figure 9b), including heterogeneous droplets (inset of Figure 9b). The endoplasmic reticulum was expanded (Figure 9b,c), and autophagosomes of various sizes were frequently found (Figure 9c–e), along with numerous primary and secondary lysosomes (Figure 9a,c–g) in both Caco-2 cells (Figure 9b) and the other cells (Figure 9c,d). But mitochondria were the most affected organelles in all three cell types. Many still had normal ultrastructure, but others showed changes that ranged from a moderate reduction of cristae to the total absence of cristae from an electron-lucent matrix (Figure 9a–f).

In some Caco-2 cells, all mitochondria were degenerated (Figure 9g), and also a high number of cells in this group were necrosed (Figure 9h), with disrupted plasma membrane and intact organelles visible, including ballooned mitochondria with cristae still visible and an expanded endoplasmic reticulum.

#### 2.8.7. MNPs 24 h Group and MNPs-MH 24 h Group

In the spheroids incubated with MNPs for 24 h, large amounts of MNPs were found relatively grouped in the cytosol of the Caco-2 cells (Figure S8a,b), occupying important volumes of the cytoplasm. In some cells, the MNPs were also identified inside still intact endosomes, or in endosomes whose membrane began to disintegrate (Figure S8a). A reduction of the size and number of microvilli and a slight mitochondrial polymorphism were identified as ultrastructural changes in the Caco-2 cells (Figure S8a,b).

In rare cells, a low number of mitochondria were ballooned, containing electron-lucent matrix (Figure S8b). An interesting aspect, noted for all groups of spheroids incubated in various conditions with MNPs (with or without Mel), was the absence of the nanoparticles in the other two types of cells forming the spheroids.

MH exposure accentuated these reactions (Figure S8c,d), some mitochondria showing a lobated aspect (Figure S8d). Most mitochondria in Caco-2 cells had fewer cristae with a normal or modified aspect (Figure S8c,d). Many other mitochondria were completely devoid of cristae, containing a relatively dense granular matrix (Figure S8d). Some cells also had very large secondary lysosomes (Figure S8c).

#### 2.8.8. MNPs 72 h Group and MNPs-MH 72 h Group

The spheroids incubated with MNPs for 72 h had a similar ultrastructure as mentioned for the 24 h incubation (Figure S8e,f). However, a tendency of Caco-2 cells to separate from the neighboring cells was noted (Figure S8f). Mitochondria were the most affected organelles. They contained either no cristae and a dense granular matrix (Figure S8e,f) or fewer cristae visible on the background of a rarefied matrix (Figure S8f).

MH exposure resulted in accentuated separation of cells (Figure S8g,h) and a generalized response of mitochondria in all types of cells. In Caco-2 cells, almost all mitochondria had a rarefied matrix and a variable number of cristae with an abnormal pattern, and in many cases, with abnormal shapes (Figure S8g,h). Rare lipid droplets were heterogeneous (Figure S8g), while in most cases, they preserved a normal aspect (Figure S8h).

#### 2.8.9. MNPs-Mel 24 h Group and MNPs-Mel-MH 24 h Group

In the spheroids incubated with MNPs-Mel for 24 h, the cells preserved their aspect and adherence. An increased number of primary and secondary lysosomes was found in Caco-2 cells, sometimes of large sizes (Figure S9a), as well as of small (Figure S9b) or large (Figure S9c) autophagosomes. The lipid content (including in the nucleus) was reduced, and the droplets had a homogeneous aspect (Figure S9c). Some mitochondria preserved in rare Caco-2 cells a normal aspect (Figure S9d), but, in most cells, the mitochondria showed a large diversity of changes (Figure S9a–h). Thus, ring-shaped mitochondria (Figure S9d,e), and with other bizarre shapes (Figure S9d,e), and mitochondria with irregular outlines (Figure S9f,g) were found. Concerning their inner structure, a general reduction in the number of their cristae was noted. The remaining visible cristae were normal (Figure S9b,d–f) or circular (Figure S9b–e,h). Additionally, many mitochondria were ballooned and contained a rarefied matrix (Figure S9a–h). Some Caco-2 cells were clearly more affected than others, but still closely attached to each other, and preserving the normal number and aspect of microvilli (Figure S9g). In the more damaged cells, almost all mitochondria were degenerated, with a few or no cristae and electron-lucent matrix (Figure S9h), while the other cellular components (nuclei and organelles) had normal aspects.

MH exposure reduced the adherence of some Caco-2 cells to each other and to the subjacent cells (Figure S10a), sometimes appearing more prominent at the surface of the spheroids (Figure S10b). Many other Caco-2 cells still remained closely attached to each other (Figure S10c,d). However, the microvilli were absent (Figure S10b), or present in a very low number at the apical region of Caco-2 cells (Figure S10c,d). The lipid content was low to moderate (Figure S10a–h), with homogeneous droplets, in general small, but sometimes relatively large, of over 2 µm (Figure S10e). Lysosomes were found in small numbers (Figure S10a,c,e,h), but also some of unusually long shapes were identified (Figure S10h). Large autophagosomes (Figure S10b), or smaller ones (Figure S10b,c,e,h), were present in these cells. As for the incubation without MH exposure, the mitochondria were again the most affected organelles, mainly in the Caco-2 cells. Most of them were ballooned, with no visible cristae and granular matrix (Figure S10a–g). Other mitochondria showed only several normal cristae (Figure S10a–d,f,g), or modified cristae (Figure S10a,b,d) and very rare mitochondria had a normal ultrastructure (Figure S10f). The mitochondria that were not ballooned were polymorphous, some of them with lobated (Figure S10c,f) or ring shapes (Figure S10d,g), or were very long, exceeding 3 µm (Figure S10e). Some degenerated mitochondria contained electron-dense inclusions within their otherwise rarefied matrix (Figure S10f,g). Some of the examined Caco-2 cells suffered deeper ultrastructural alterations, with cytoplasmic rarefaction and apparently without mitochondria, but with electron-lucent vesicles resulted from extreme mitochondrial degeneration (Figure S10h).

#### 2.8.10. MNPs-Mel 72 h Group and MNPs-Mel-MH 72 h Group

**Figure 10:**
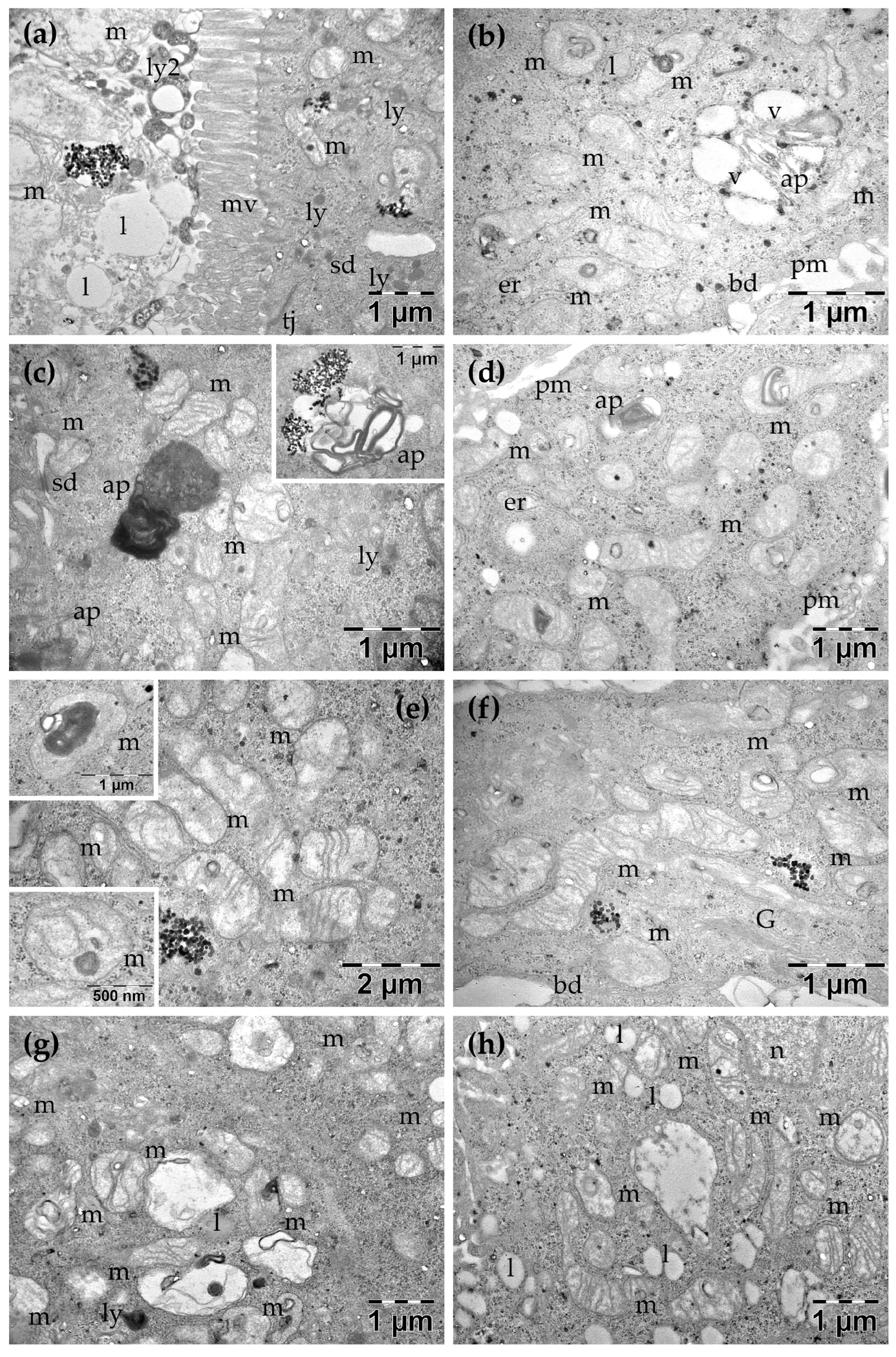
(**a**–**h**) Representative TEM images of cells in spheroids from MNPs-Mel 72 h group. ap: autophagosome; bd: belt desmosome; er: endoplasmic reticulum; G: Golgi apparatus; l: lipid droplets; ly: lysosome; m: mitochondria; mv: microvilli; pm: plasma membrane; sd: spot desmosome; tj: tight junction; v: vesicle.

Spheroid incubation with MNPs-Mel for 72 h resulted in the total destruction of many Caco-2 cells (Figure 10a). Such cells had disrupted plasma membrane and organelles (including ballooned mitochondria) and lipid droplets dispersed in the extracellular space. The other intact remaining Caco-2 cells preserved a normal aspect (number and size) of the microvilli at the apical pole (Figure 10a) but underwent important ultrastructural changes in their organelles (Figure 10a–h). The number of internalized MNPs-Mel was variable in the examined Caco-2 cells, and they were mainly present in the cytosol, more or less grouped (Figure 10a,c,e,f), and sometimes in autophagosomes (inset of Figure 10c). Some of the Caco-2 cells preserved normal adherence (Figure 10a), but in most cases, a tendency to separate from each other and from the other types of cells was noted (Figure 10b,d,f). A low number of small primary lysosomes was found (Figure 10a,c,g). On the other hand, many autophagosomes of different sizes and aspects were observed (Figure 10b–d), some exceeding 1 µm in diameter (inset of Figure 10c). The lipid content was reduced and the droplets were small and homogeneous (Figure 10b,g,h), and in some cells, electron-lucent vesicles were observed (Figure 10b).

Finally, the mitochondria in the Caco-2 cells of this group suffered the highest diversity of ultrastructural changes among all studied groups. In general, the mitochondria had round/oval shapes (Figure 10a–h), and some of them were very long (Figure 10c–f,h), or polymorphic and/or with irregular contour (Figure 10b–h). The majority of mitochondria contained normal or slightly reduced numbers of cristae, with a normal aspect (Figure 10a,c,g). Nevertheless, in many mitochondria, cristae with abnormal shapes were identified, in general circular (Figure 10b–h) or, in some cases, multiple cristae arranged concentrically (Figure 10b,d–g). A number of mitochondria with unusual electron-dense inclusions were present in the Caco-2 cells (insets of Figure 10e,g). Even though most mitochondria preserved a normal aspect of their matrix, in several ballooned mitochondria, the matrix was electron-lucent (Figure 10a,c,g,h). This aspect, along with their degenerated cristae, made such mitochondria look like autophagosomes (Figure 10g). Also, a low number of mitochondria were ballooned, with an electron-lucent matrix and completely devoid of cristae (Figure 10g,h).

**Figure 11:**
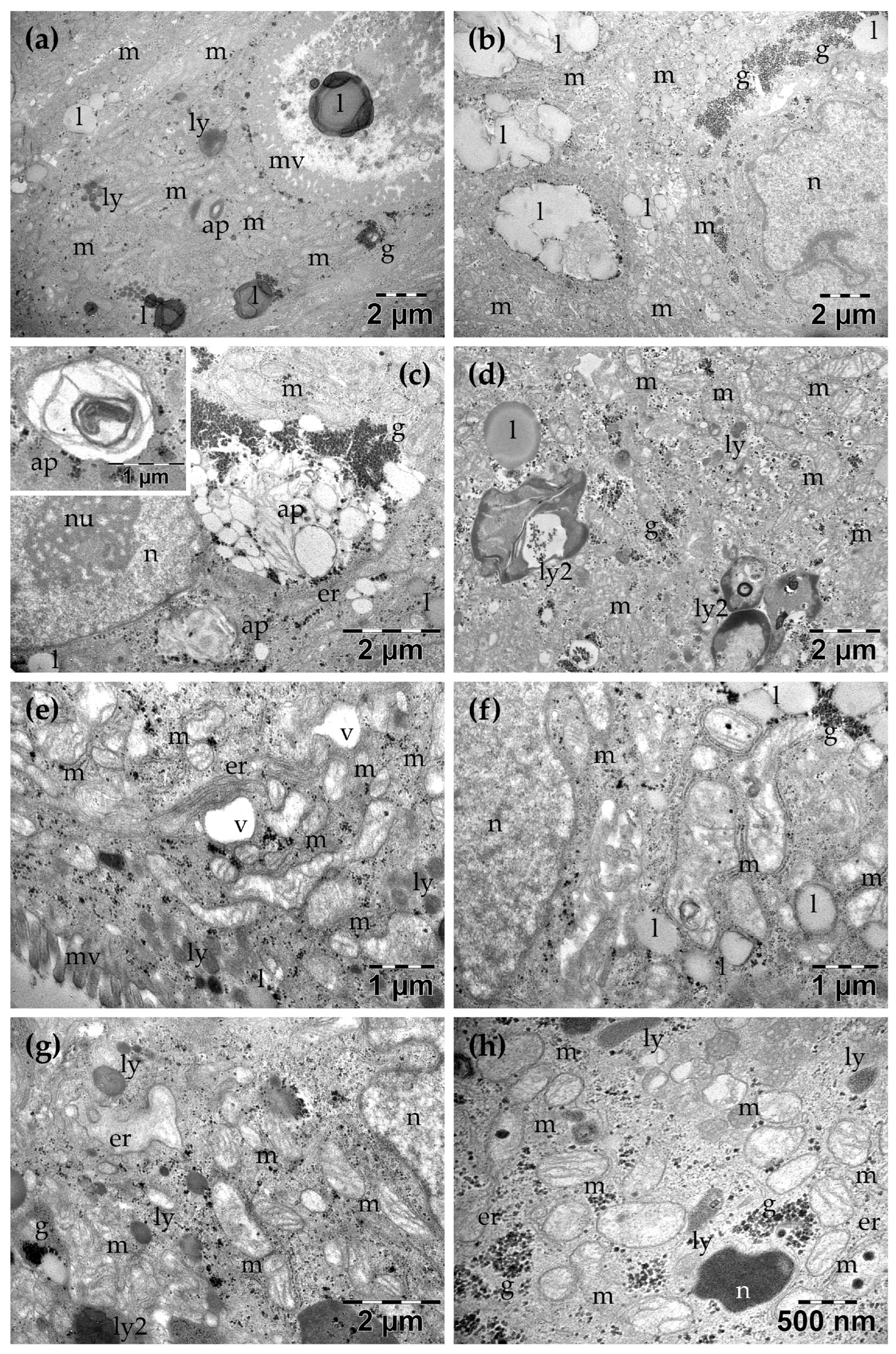
(**a**–**h**) Representative TEM images of cells in spheroids from MNPs-Mel-MH 72 h group. ap: autophagosome; er: endoplasmic reticulum; g: glycogen; l: lipid droplets; ly: lysosome; ly2: secondary lysosome; m: mitochondria; mv: microvilli; n: nucleus; v: vesicle.

MH exposure after the 72 h incubation with MNPs-Mel killed many Caco-2 cells, their debris being dispersed in the extracellular space (Figure 11a). The remaining intact cells contained numerous lipid droplets with different appearances: round, electron-dense and with a heterogeneous aspect (Figure 11a), polymorphic, less electron-dense and a heterogeneous (larger than 5 µm) or homogeneous aspect (Figure 11b), or intermediate forms between the two (Figure 11a–f). Most cells also contained autophagosomes, including very large ones (Figure 11a,c), and many primary and secondary lysosomes, the latter also of large sizes (Figure 11d,g); additionally, unusually long primary lysosomes were found (Figure 11h). The endoplasmic reticulum was in general very expanded (Figure 11c,g,h). Mitochondria with a normal ultrastructure were observed in rare cells (Figure 11c,d), but almost all of them were polymorphic (Figure 11c–g), with fewer and/or abnormal cristae (Figure 11c–h), and with different degrees of matrix rarefaction (Figure 11c–h). Moreover, many mitochondria were completely devoid of cristae (Figure 11e–h). Rare mitochondria with few or no cristae contained large electron dense inclusions (Figure 11h).

## 3. Discussion

Experimental models resulted from three-dimensional culture or co-culture of cells in spheroids represent a step forward for in vitro testing of active agents. In the current paper, this model, based on colorectal cancer cells, was successfully used to investigate the antitumor effects of melittin, administrated either free or as melittin-functionalized magnetic nanoparticles (at matched dose of Mel), in several experimental conditions, including exposure to magnetic hyperthermia.

Preliminary testing on monocultures proved the ability of the cells of interest to internalize the MNPs-Mel, as a starting point for the main phase of experimentation. The spheroids containing Caco-2 cells, BJ fibroblasts and HUVECs, present at a 3:1:1 ratio within a collagen-I matrix, had the expected spheric shape and showed a well-structured organization, with Caco-2 cells delineating their periphery, and covering thus the other cells. Moreover, the cellular distribution in our spheroids, as well as the presence of cell junction between Caco-2 cells and between them and the other cells, mimics to a certain extent the behavior and co-ordination of cells in tissues or organs. Thus, the understanding of MNPs-Mel dynamics and their effects as primary essential aspects when intending to design in vivo experiments is facilitated.

Spheroid architecture provides context for interpreting the biochemical response. The peripheral epithelial organization and cell–cell contacts described by microscopy indicate a structured multicellular model, while the treatment-associated loss of adhesion, cellular disruption and organelle damage show that its structural state changed with exposure. MTS and ATP signals consequently integrate both the amount of metabolically active cellular material and the metabolic state of those cells. A decrease can therefore reflect functional suppression, loss of viable cells or both. Reading these assays together with TEM gives a more informative account of treatment response than interpreting either metabolic signal alone.

Rather than evaluating cellular cytotoxicity alone, we integrated here metabolic activity, potential interference with angiogenesis, mechanisms of cell death and TEM examination to determine whether MNPs-Mel formulation has different impact on the magnitude of spheroid-cell forming cells reaction to Mel.

Since the spheroids could not be probed, nor photographed in IR at a satisfactory resolution, the reported temperatures represent mean values and do not capture localized heating generated by MNPs. We acknowledge that these temperature increases are modest and do not reach the hyperthermic range typically required for effective therapy. The small bulk temperature increase (~1 °C, 38.1–38.6 °C vs. 37.6 °C control) observed during spheroid exposure is attributable to several factors: (1) The amount of MNPs internalized by cells in spheroids was significantly lower than that present in colloidal suspensions, reducing their overall heating capacity. (2) Only the peripheral layers of spheroids internalized MNPs, and therefore MH produced a two-direction thermal gradient (towards the center of the spheroid, and towards the external liquid medium), resulting in more accentuated effects in the Caco-2 cells. (3) Once internalized, many MNPs were confined within endosomes, which limited their magnetic relaxation and thus their heating efficiency. However, this moderate hyperthermia contributed to the Mel release from the MNPs (see Figures S3 and S5), accentuating the cytotoxic effects recorded for MNPs-Mel in the absence of MH. Importantly, the local temperature at the nanoparticle surface is expected to be considerably higher than the bulk temperature; however, due to thermal dissipation into the surrounding medium, only a fraction of this localized heat is detected by the fiber-optic probe. Thus, the bulk measurement likely underestimates the actual thermal dose experienced at the cellular level. In our experiments, we believe the AMF effects arise from both mechanisms acting synergically: (1) the heat dissipated by the magnetic nanoparticles under the AMF, which locally sensitizes the cells (even though the bulk temperature rise is modest), and (2) field-triggered release of Mel from the MNP surface, which is enhanced by the localized heating at the nanoparticle level. Since the bulk temperature increase of ~1 °C does not meet the classical definition of hyperthermia (typically ≥ 42 °C at the tissue level), we refer to the observed effects as being magnetic hyperthermia-mediated intracellular heating combined with magneto-chemotherapeutic (Mel release) effects, rather than purely thermal ablation.

The five significant treatment × time interactions established that the response of the tri-cellular spheroids changed between 24 and 72 h and the cells showed a different behavior as that reported for testing of individual cultured cells [7]. The experiment therefore describes a dynamic, integrated response of tumor cells (prevalent) and two normal cell types. None of the assays separated the three cell populations. The changes in viability reduction, treatment toxicity, angiogenesis markers and apoptosis consequently cannot be assigned specifically to tumor cells, fibroblasts, or endothelial cells.

All five treatments reduced the cell viability relative to the untreated spheroids at both time points, demonstrating lower viability across the complete multicellular structure. MTS and ATP are viability/toxicity-associated metabolic readouts that integrate viable-cell abundance and metabolic state across all three cell types.

Unloaded MNPs produced the highest viability levels among the treated groups at 24 and 72 h, indicating the least pronounced metabolic effect. MNPs-MH produced lower MTS and ATP signals than unloaded MNPs at both intervals, although the ATP separation was smaller at 72 h. The ATP response to AMF was less pronounced with unloaded MNPs than with MNPs-Mel, indicating that the effect of AMF depends on the formulation. The unloaded nanoparticles lack the membrane-active peptide and therefore need not reproduce the metabolic response of the combined system. At the later interval, preservation of the ATP pool in surviving cells may also reduce the apparent separation between the unloaded-MNP groups, even when MTS and TEM reveal treatment-associated changes. The present findings show that the strongest ATP suppression occurred when AMF exposure was combined with Mel-functionalized nanoparticles.

Melittin functionalization produced a distinct metabolic profile. MNPs-Mel yielded lower viability than free Mel and unloaded MNPs at both 24 and 72 h, with a particularly large separation at 72 h. This demonstrates a stronger formulation-level reduction in whole-spheroid MTS-reducing activity after nanoparticle presentation of melittin. The finding does not identify which cellular compartment was preferentially affected and does not by itself demonstrate selective delivery to Caco-2 cells.

Both MNPs-MH and MNPs-Mel-MH produced lower mean MTS and ATP signals than Control-MH at both intervals. At 72 h, their MTS responses were 66.08% and 8.80%, respectively, compared with 83.00% for Control-MH; the corresponding ATP responses were 127.68% and 24.08%, compared with 155.00%. All percentages use the untreated 24 h control as the reference. Inclusion of an AMF-only control allowed the response to field exposure to be evaluated separately from the additional effects associated with nanoparticle-containing treatments. The lower MTS-reducing activity in Control-MH may reflect a change in cellular metabolic state following AMF exposure. MTS reduction depends on cellular reducing activity as well as the amount of viable biomass, so a lower signal need not represent a proportional decrease in viable-cell number. The broadly preserved spheroid architecture on TEM is consistent with a metabolic component to the response, while the accompanying decrease in ATP suggests altered cellular energy status. This corresponds to the study of Iacovita et al., who reported no significant decrease in viability following AMF exposure alone in A549 cells and human gingival fibroblasts under their experimental conditions [19]. Both MNPs-MH and MNPs-Mel-MH produced additional reductions related to Control-MH, supporting an effect associated with the nanoparticle-containing treatments beyond the response to AMF exposure alone. ATP level and viability did not change in parallel after MNPs-Mel treatment. At 24 h, MNPs-Mel and free Mel produced comparable ATP-associated luminescence despite a lower viability signal with MNPs-Mel. At 72 h, ATP was higher with MNPs-Mel than with free Mel, while viability remained much lower with MNPs-Mel. These divergent patterns show that the two assays captured different aspects of the multicellular response.

MNPs-Mel-MH produced the lowest ATP at both timepoints. Relative to MNPs-Mel, the combined treatment sharply reduced ATP at 24 and 72 h. The viability profile was temporally different: at 24 h, MNPs-Mel-MH retained a higher MTS signal than MNPs-Mel, whereas at 72 h, it produced the lowest viability from all groups. These results show a time-dependent divergence between the two metabolic readouts. Because the assays were performed on separate spheroids, the data do not establish whether one response preceded or caused the other.

The reductions produced by free Mel in both viability and ATP are qualitatively consistent with the dose-dependent loss of viability and rapid membrane disruption reported by Soliman et al. in COLO205 and HCT-15 colorectal cancer monolayers [20]. The studies are not directly comparable in effect magnitude because the published work used different colorectal cell lines, Mel exposures, and a two-dimensional monoculture, whereas the present endpoints integrate three cell types within a collagen-I spheroid.

A particularly relevant comparison is our Caco-2/BJ monolayer study, in which Mel-functionalized Fe_3_O_4_ nanoparticles reduced viability-associated and ATP signals, and MH intensified the response and increased caspase-9 [7]. The present tri-cellular results reproduce the same overall direction at the whole-spheroid level: MNPs-Mel strongly suppressed cell viability, and MNPs-Mel-MH produced the lowest ATP signal at both times and the lowest MTS signal at 72 h. The current findings therefore show that the formulation-level response mostly persists in a 3D environment containing malignant epithelial, and stromal cells: normal fibroblast and endothelial compartments.

The enhanced effect of MNPs-Mel-MH is also consistent with three-dimensional magnetic-hyperthermia work by Stocke et al., who observed substantial damage after alternating-field exposure of magnetic nanoparticle-containing multicellular cancer-stromal tissue analogues, while the magnetic material alone produced comparatively limited damage [21]. MTS-reducing activity and ATP-associated luminescence assess different aspects of cellular metabolism. MTS reports cellular reducing activity, whereas the ATP signal reflects the ATP pool present at sampling, which depends on both ATP generation and consumption. At 24 h, MNPs-Mel-MH therefore could retain more MTS-reducing activity than MNPs-Mel alone while showing a much lower ATP signal. This pattern is compatible with pronounced energetic impairment despite residually reducing activity in surviving cells. At 72 h, the combined treatment produced the lowest values in both assays, consistent with more extensive metabolic and structural injury. Conversely, the relatively preserved ATP signal with MNPs-Mel alone, despite marked MTS suppression, may reflect a different metabolic state or composition of the surviving cell population. Because the assays were performed on separate spheroids, these patterns describe complementary endpoints rather than a demonstrated sequence of cellular events.

Concerning the angiogenetic markers, VEGF concentration did not mirror the metabolic endpoints. At 24 h, free Mel, MNPs, and MNPs-Mel-MH reduced VEGF relative to the control, whereas MNPs-Mel and MNPs-MH did not. At 72 h, all groups showed lower VEGF levels compared to controls, although the reduction with MNPs-Mel alone was comparatively small. The pronounced MTS suppression produced by MNPs-Mel was therefore not accompanied by a proportional reduction in VEGF. This can be due to an initial selective killing of endothelial cells (24 h) more susceptible to Mel [7], responsible for vascularization, leading to increased hypoxia, which generated higher amounts of HIF1α, leading to downstream secretion of VEGF from the surviving cancer and endothelial cells. At 72 h, following a higher cellular-induced death, the HIF-1α response was diminished and led to a lower angiogenetic response measured by decreased VEGF. Therefore, the two phases of the angiogenetic response were linked to the amplitude of the hypoxia level and the type of surviving cells. The 72 h inhibition of angiogenesis is a valuable addition to the therapy since it addresses the vascularization of the tumor responsible for growth and eventually metastasis. The MH addition to MNPs-Mel enhances this anti-angiogenetic response and prolongs the activity of the Mel initial killing by adding the associated increased intracellular release of Mel from the MNPs-Mel.

The clearest formulation-associated VEGF response occurred when MH was applied to MNPs-Mel. VEGF decreased from 201.02 to 47.81 pg/mL at 24 h and from 296.11 to 102.69 pg/mL at 72 h. In contrast, MH changed the unloaded-MNP response in opposite directions across time: VEGF was higher with MNPs-MH than with MNPs at 24 h but lower at 72 h. These findings indicate that the VEGF response depended jointly on nanoparticle functionalization, MH exposure, and assessment time.

The reduction in VEGF produced by free Mel at both timepoints agrees with Wang et al., who reported that Mel reduced VEGF secretion and angiogenic tube formation in HUVEC experiments performed alongside colorectal cancer models [16]. MNPs-Mel alone did not reproduce an equally strong VEGF decrease in the present spheroids, particularly at 24 h, despite its pronounced MTS suppression. By contrast, application of MH to MNPs-Mel restored and intensified the VEGF-lowering response. This comparison indicates that nanoparticle presentation and AMF exposure altered the relationship between metabolic suppression and the VEGF signal rather than merely reproducing the effect of free Mel.

VEGF is a major regulator of endothelial responses, and HUVECs can form angiogenic structures in response to tumor-derived VEGF in three-dimensional co-culture systems [11]. The particularly low VEGF values after MNPs-Mel-MH coincided with the lowest ATP signal and a substantial reduction in MTS-reducing activity.

HIF-1α showed a distinct temporal pattern. Free Mel and MNPs-Mel-MH increased HIF-1α at 24 h, whereas MNPs, MNPs-Mel, and MNPs-MH decreased it. At 72 h, HIF-1α remained elevated with free Mel and was elevated with MNPs-MH; MNPs, MNPs-Mel, and MNPs-Mel-MH were not different from the control. For the combined treatment, the increase from 0.0327 ng/mL with MNPs-Mel to 0.1141 ng/mL with MNPs-Mel-MH at 24 h was not sustained at 72 h.

HIF-1α can activate VEGF transcription under hypoxic conditions [12], but HIF-1α and VEGF were not directly proportional in this experiment. This differs from the coupled suppression described by Shin et al. in epidermal-growth-factor-stimulated CaSki cervical cancer cells, where Mel reduced HIF-1α accumulation, VEGF secretion, and angiogenic activity [22]. Here, free Mel lowered VEGF but increased HIF-1α at both times, while MNPs-Mel-MH combined low VEGF with a transient HIF-1α increase at 24 h. The measured HIF-1α and VEGF concentrations need not vary in parallel because their production, release and turnover differ. VEGF is a secreted mediator, whereas HIF-1α normally functions within cells; its presence in culture supernatant may also be influenced by release from stressed or injured cells. The higher HIF-1α concentration with MNPs-Mel-MH at 24 h, together with lower VEGF, is therefore compatible with an early stress-associated response occurring alongside reduced VEGF availability. The absence of a sustained HIF-1α increase at 72 h may reflect a changed state or composition of the surviving population and different extracellular protein kinetics. This interpretation attempts an explanation of the temporal dissociation without assuming that supernatant HIF-1α directly represents intracellular HIF activity or the oxygen status of the spheroid.

The three-dimensional context is relevant to this divergence. Shweiki et al. demonstrated that multicellular spheroids can develop spatially restricted hypoxic and glucose-deprived regions that promote VEGF expression [4]. Different kinetics of HIF-1α accumulation, VEGF synthesis and release, and loss of metabolically active spheroid mass, may all contribute to the observed dissociation.

The ELISA measured immunoreactive caspase-9 concentration in the collected culture medium. This concentration reflects the protein available extracellularly at sampling and can be influenced by cellular abundance, release and protein turnover. The higher caspase-9 values with several treatments are consistent with an injury-associated response involving a protein of the intrinsic apoptotic pathway. They should be interpreted alongside the mitochondrial and cellular changes on TEM, rather than as a direct measure of caspase activation or the number of apoptotic cells.

MNPs-Mel no longer increased caspase-9 relative to control at 72 h despite persistent MTS suppression and marked ultrastructural injury. One plausible explanation is that the later sample reflects a different balance between protein release from injured cells, the remaining cellular population and extracellular protein turnover. Consequently, a control-like caspase-9 concentration can coexist with substantial treatment-associated damage and does not indicate recovery of the spheroid. In contrast, MNPs-Mel-MH produced elevated caspase-9 at both intervals, reaching 0.444 ng/mL at 72 h, alongside the lowest MTS and ATP signals. This combination is compatible with more extensive injury in the combined-treatment group, without requiring every damaged cell to follow the same death pathway.

The increase produced by free Mel at both times parallels the findings of Wang et al. in HCT116 and HT29 colorectal cancer cells, where Mel increased apoptosis and engaged a mitochondria-associated pathway involving mitochondrial reactive oxygen species, loss of membrane potential, cytochrome-c release, and caspase-9 [16].

MNPs-Mel produced more caspase-9 than unloaded MNPs at 24 h but not at 72 h. Its sustained reduction of MTS activity therefore occurred without a sustained increase in total caspase-9 concentration. By contrast, adding MH to MNPs-Mel increased caspase-9 at both time points, from 0.334 to 0.404 ng/mL at 24 h and from 0.226 to 0.444 ng/mL at 72 h. The latter value was the highest numerical caspase-9 concentration observed at 72 h and coincided with the lowest MTS and ATP signals.

This MH-associated caspase-9 response is directionally consistent with the earlier Caco-2 monolayer results for the same Mel-functionalized Fe_3_O_4_ platform, but with higher response of BJ cells at 24 h and of Caco-2 cells at 72 h [7]. The present increase is observed in a mixed spheroid and therefore extends the formulation-level pattern to a more complex experimental system, while remaining an aggregate response that cannot be attributed specifically to Caco-2 cells.

Caco-2 cells may contribute substantially to the aggregate treatment response because they comprised 60% of the initial cell mixture, formed the recognizable peripheral epithelial layers and displayed pronounced mitochondrial and cellular changes on TEM. Their position also places them in direct contact with the surrounding treatment medium. These observations provide a biological basis for considering an important Caco-2 contribution. However, the initial seeding ratio does not determine each population’s contribution at the time of sampling, and BJ fibroblasts and HUVECs may also contribute to the measured caspase-9 and metabolic signals. The biochemical results are therefore interpreted as the integrated response of the tricellular spheroid.

The results reported here are consistent with those obtained by us for monocultures of Caco-2 cells [7]. Our previous A375/HaCaT study provides evidence of apoptosis-associated responses to related treatments in monolayer cultures [23], while the present findings describe the integrated biochemical and structural response of tricellular spheroids. In addition, Soman et al. showed concentration-dependent elevation of mitochondrial cytochrome c release followed by increase in early and late apoptosis in C32 melanoma cells treated with Mel-functionalized nanoparticles, and limited damage of plasma-membrane [24]. Our results also align with the literature data reporting that normal cells possess lower sensitivity to Mel than tumor cells [7,24,25].

Transmission electron microscopy is a laborious but rewarding technique, essential for characterization of nanomaterials, in assessing the intracellular presence of nanomaterials as well as their fine effects at subcellular level. It also completes and explains molecular reactions revealed by the other methods and helps in correlating various results, thus being a useful tool for the overall understanding of the mechanisms involved. Therefore, TEM was used here to observe the size and to precisely measure the MNPs, and to describe the structure and organization of spheroids, including the establishment and type of cell junctions between the cells. Additionally, it was used to demonstrate the actual internalization and location of the MNPs-Mel, in both monoculture and spheroid models, and to reveal the ultrastructural changes in relevant organelles generated by the various tested agents in the spheroid-forming cells.

Incubation of spheroids with Mel at 2.5 µg/mL confirmed the time-dependent reactions of Caco-2 cells reported by the molecular results. If only limited changes at the plasma membrane, mitochondria or lipid droplets level were noted at 24 h, the prolonged exposure resulted in reduced adhesivity, and reactions of all cellular structures: accentuated mitochondrial polymorphism and degeneration, expanded endoplasmic reticulum, stimulation of cellular digestion, including autophagy, increased lipidic content in cytoplasm and nucleus. The longer presence of Mel resulted in many necrotic cells as well. These results are consistent with those previously reported for Caco-2 monoculture [7].

Following the incubation of spheroids with MNPs (50 µg/mL), only some Caco-2 cells contained nanoparticles. In this experimental setup, internalization was limited by the cellular polarization and the presence of numerous microvilli at the apical pole exposed to the MNPs-Mel containing culture medium. Additionally, the presence of intact tight junctions between Caco-2 cells restricted the direct passage of nanoparticles towards the spheroid core cells. Consequently, TEM examination showed low-level cellular reactions, limited to mitochondrial ballooning, and tendency towards cellular separation after long-time incubation with MNPs and MH exposure. The prolonged contact with nanoparticles theoretically increased the probability of cells to internalize them, supposition sustained here by the molecular results, as well as by the ultrastructural changes documented at subcellular level. Future studies will demonstrate it by precise dosing of iron atoms in the cells. Yet, testing of bare MNPs in the four experimental conditions was required to discriminate their effects from the actual effects of the Mel delivered intracellularly by the MNPs.

No nanoparticle-containing lysosomes, and very rare intact endosomes with MNPs (or MNPs-Mel), were found on our ultrathin sections. This suggests the occurrence of two distinct mechanisms involved. The first one, and probably the most important, was the mechanical rupture of the endosomal membrane by the MNPs. The second one consisted of a direct toxic effect of Mel vehiculated by MNPs on the endosomal membrane, with quick release of MNPs-Mel in the cytosol. However, either mechanism could also explain the absence of transcytosis, and implicitly the absence of MNPs (with or without Mel) from the surroundings of the inner cells, and from their cytoplasm, despite their high ability to internalize MNPs-Mel, as demonstrated in Section 2.8.2.

MNPs-Mel released from endosomes during the two incubation intervals interacted with various organelles and triggered time-differentiated effects. At 24 h, mitochondria were again the most affected organelles, showing a very interesting polymorphism, mitochondrial reactions being amplified by MH exposure, along with a tendency of Caco-2 cells to separate from each other and from the other cells. At 72 h, massive and generalized cellular and subcellular alterations were produced by MNPs-Mel. The autophagy activation constitutes supplementary evidence supporting the deep alteration of cellular components in this group. These generalized changes were further accentuated by the MH exposure, only rare organelles with normal aspect being found; most mitochondria were devoid of cristae and extremely polymorphic. The impact of MH on mitochondria ultrastructure in the spheroids treated with MNPs-Mel for 24 and 72 h explained the low amount of ATP measured here (reduced to 23% and to 14% respectively, as compared with the corresponding controls). Again, these results correlate with those reported for testing in monoculture [7]. Moreover, suffering mitochondria in the various treatment groups impacted the metabolic viability of cells, and caspase-9 levels both in 2D Caco-2 cells culture [7], and here in spheroid model.

The effects reported in this paper are mainly generated by the presence of Mel in the culture media and could be explained by the interesting properties of this membrane-targeting peptide. When spheroids were incubated with free Mel, the molecule was able to directly interact with plasma membrane of Caco-2 cells, and it remains possible that a low amount diffused among the other subjacent cells consecutive to adherent junctions’ destabilization. Mel is an amphipathic molecule with high affinity for biological membranes due to the 20 hydrophobic amino acids residues forming the N-terminal segment [26]. The electrostatic interactions also participate in Mel adsorption at the membrane’s surface, when present in a low concentration [27]. At higher concentrations, and once inserted in a membrane, it assembles as tetramers, with α-helix conformation involving 24 amino acid residues [26,28,29,30]. Here, the peptide perturbs lipid organization and mobility [31], aggregates the intrinsic membrane proteins [32], and can even permeate the bilayer by removing lipid–protein aggregates surrounded by Mel [33]. Depending on concentration, lipid composition, cholesterol content and membrane charge, Mel adopts a partially transmembrane position [33,34]. It will organize, by different proposed mechanisms [29,30,35,36,37], pores of 2.5–3 nm [38], with selective and measurable permeability for water [39,40], in which residues Ile^17^-Ser^18^-Trp^19^ play a key role, as showed by Berneche et al. [41]. At even higher concentrations, Mel is responsible for membrane swelling, blebbing and disruption [20] according to a detergent-like mechanism [29,30,35,36,37]. Considering these membrane-related effects, Mel was tested for anticancer effects against different types of cells [42,43,44,45,46,47]. Moreover, literature data shows that Mel arrived intracellularly possesses anticancer properties based on other molecular mechanisms including signaling pathways [48] or caspase-dependent apoptosis [7,23,49,50].

It was intended to compare here the effects of low doses of free Mel added to the culture media with the effects triggered by equivalent doses of Mel adsorbed at the surface of the MNPs with which the spheroids were incubated. And there were found indeed more accentuated and complex molecular and ultrastructural results when using MNPs-Mel than with free Mel, even though the experiments were performed in the same conditions. A simplistic explanation could reside in the different mass of the tested agents. Dispersion of Mel into the culture media favored interaction of a limited number of molecules with the cells at spheroid surface, an even lower amount of Mel arriving in contact with the inner cells. On the other hand, the presence of certain number of Mel molecules at the MNP surface accelerated the contact of cells with a much higher number of Mel molecules brought by their nanoparticle transporters through gravity (or side by side by the continuous rocking) towards the bottom of the culture plates where the spheroids were placed. As in the case of free Mel, many MNPs-Mel remained outside the cells, as the TEM images showed. However, when MNPs-Mel arrived in contact with plasma membrane, they could act by two complementary mechanisms. The first one in chronology was achieved by direct contact of Mel hydrophobic extremities (distally placed around the MNP core) with plasma membrane, and subsequent permeation or perturbation of the lipid–protein bilayer. The second, and the most relevant one for our study, resulted from MNP internalization in a high or a very high number. Endosomes contained large amounts of MNPs-Mel, and shortly after endosomal formation, they were ruptured by mechanical forces (as in the case of bare MNPs), but with supplementary intervention of Mel-adsorbed molecules. This way, a certain amount of Mel arrived within the cytosol and in contact with various cellular structures to produce the limited effects found in the examined cells. In the next phase of the experiments, intervention of the alternative magnetic field contributed both to the accelerated releasing of MNPs-Mel from endosomes, through the rupture of their surrounding membrane, and to the release of more Mel from MNPs inside and outside the endosomes. Additionally, a third possible consequence of the applied MH concerned the MNPs-Mel still attached to the plasma membrane surface, after the spheroid washing that preceded the MH exposure. They could thus augment the damages of this lipidic bilayer, with potential consequences for the intracellular alterations. This is why the most severe molecular changes and ultrastructural degenerations were identified in the MNPs-Mel-MH group of spheroids. Mitochondria were among the most affected organelles in this experimental setting (many with extensive depletion of cristae), confirming the lower capacity to produce ATP, and sustaining the high levels measured for caspase-9. On the other hand, the reduced amount of ATP resulted in our study from the lower number of cells surviving the experimental treatments, as shown by the viability tests. Of course, the high number of necrosed cells identified in this group, along with the overall reduced metabolic viability of remaining functional cells also explain the low amount of ATP reported for the MNPs-Mel-MH group. Altered or degenerated mitochondria were also previously reported when testing in vitro the effects of free Mel [51], or other nanomaterials [52,53].

The relevance of MNPs as a platform for Mel delivery in the current study comes from their ability to deliver important amounts of Mel inside the cells, to release it and to increase the local temperature by several degrees [7] when exposed to an alternating magnetic field, potentially triggering heat shock responses. In certain situations, magnetic guidance also helps the precise targeting of the MNPs. On the other hand, Farahavr et al. showed that externally generated heat by near-infrared photothermal exposure of functionalized gold nanocages increased the cytotoxic activity of Mel in A375 melanoma cells [54]. In most cases, the MNPs Fe_3_O_4_ core is surrounded by various coatings (citrate in our particular case) to prevent magnetite oxidation, aggregation or other interactions, and to provide functional groups or electric charges for attachment of the active agent (Mel in our study) [55,56,57,58]. Advantages of applying MH after intratumoral iron-oxide MNPs administration were demonstrated in clinical investigations [59,60], but the presence of Mel at the surface of the MNPs could accentuate the antitumor effects.

In therapeutic applications, Fe_3_O_4_ nanoparticles can act as carriers for drugs, proteins, peptides, and nucleic acids. Their magnetic characteristics also support functions such as magnetic localization and retention, as well as magnetic hyperthermia. Consequently, these nanoparticles can perform several complementary roles within the same nanosystem rather than functioning only as delivery vehicles [37,61,62,63,64]. This versatility is especially valuable in oncology, where treatment efficacy may be limited by insufficient accumulation at the target site, inefficient delivery, and toxicity to healthy tissues. The integration of diagnostic and therapeutic capabilities within a single structure places Fe_3_O_4_ nanoparticles within the broader field of theranostics [62,65,66,67]. Numerous surface coatings have been investigated, including polyethylene glycol, dextran, chitosan, silica, citrate, phosphonate ligands, lipids, proteins, and synthetic polymers. As the coating forms the primary interface with the biological environment, its composition plays an important role in determining cellular interactions, circulation time, persistence, and overall therapeutic performance [68,69,70,71,72].

Despite the important data provided, this study has several limitations. First, we could not clearly identify the contribution of the normal cells used to produce spheroids in the obtained molecular results, nor the mechanisms involved in this contribution. On the other hand, we could not establish the precise amount of internalized MNPs, nor whether the core spheroid-forming cells internalized MNPs or not. And we are aware that testing of two or more doses could provide additional interesting data. However, to the best of our knowledge, we report here the first molecular and ultrastructural results of antitumor effects provided by melittin in this formulation, as MNPs-Mel, with or without magnetic hyperthermia in a three-dimensional model of three-cellular spheroids.

Melittin’s known membrane activity and hemolytic potential remain relevant to systemic use, so the present results should be understood as evidence of activity in the spheroid model rather than as evidence of systemic tolerability.

## 4. Materials and Methods

### 4.1. Synthesis of MNPs, Functionalization with Melittin and Their Characterization

Synthesis, functionalization and characterization of the magnetic nanoparticles (MNPs) were extensively described in a previously published paper [7]. For the current work, we used the same batch of MNPs, the experimental part of the two studies being completed in parallel. Briefly, the MNPs were synthesized from 2 mmol of iron(III) acetylacetonate (Merck Schuchardt OHG, Hohenbrunn, Germany), 12 mmol of oleic acid (Sigma-Aldrich, Steinheim, Germany), 0.5 mL of ethylene glycol (Carl Roth GmbH, Karlsruhe, Germany), and 20 mL dibenzyl ether (Sigma-Aldrich, Steinheim, Germany) stirred for 30 min (500 rot/min) at 50 °C on a magnetic stirrer (Heidolph Instruments GmbH & Co. KG, Schwabach, Germany). The mixture was heated at 200 °C for 2 h, and at 300 °C for 1 h in an oven (Nabertherm GmbH, Lilienthal, Germany), and the formed MNPs were magnetically separated and washed with ultrasonication (3 × 15 min each) in an Elmasonic P 70 H (Elma Schmidbauer GmbH, Singen, Germany) bath sonicator. For functionalization, the MNPs were dispersed for 1 h at 80 °C in citric acid (Sigma-Aldrich, Steinheim, Germany) solution (at final concentration of 0.1 M). The MNPs were washed with ultrasonication (3 × 15 min each), magnetically separated, and dispersed in water. In the second step, to 1 mL of Mel solution containing 116.34 ± 4.41 μg Mel was added 1 mg of citrate-coated MNPs, ultrasonicated (10 min) and mechanically shaken (10 min). The MNPs-Mel were magnetically separated, and the remaining liquid was analyzed by UV-Vis spectroscopy, observing the peak at 280 nm (as well as the initial Mel solution + MNPs). This way, the concentration of Mel adsorbed was calculated at 54.57 µg Mel/mg MNPs.

The MNPs were characterized by UV-Vis with a T92+ UV-VIS spectrophotometer (PG INSTRUMENTS, Leicestershire, UK), by X-ray powder diffraction with a Bruker D8 Advance diffractometer (Bruker AXS GmbH, Karlsruhe, Germany), and the magnetic measurements with a vibrating sample magnetometer (Cryogenic Limited, London, UK). The heating efficiency was evaluated using an Easy Heat 0224 system (Ambrell, Scottsville, NY, USA). The shape and size of MNPs were evaluated using a JEOL JEM-100CX II (Jeol, Tokyo, Japan) transmission electron microscope, and the images were recorded with a MegaView G3 camera (Emsis, Münster, Germany) running with Radius 2.1 software. The diameter of MNPs was measured with Image J 1.54f (National Institute of Health, University of Wisconsin, Madison, WI, USA).

### 4.2. Cell Culture and Tumor Spheroid Model Preparation

Three human cell lines were used in this study to test their MNPs-Mel internalization abilities, to produce and grow the spheroids and to test their reactions in various experimental conditions: Caucasian colon adenocarcinoma Caco-2 (ECACC, Sigma Aldrich, Co., Heidelberg, Germany), dermal fibroblasts (BJ, ATCC CRL-2522™, Gaithersburg, MD, USA) and human umbilical vein endothelial cells (HUVECs, ECACC). Upon their purchase, they were multiplied for the first two passages to make a stock. Afterwards, the cell lines were used at first passages in our experiments. The cell cultures showed no signs of contamination, and during multiplication and experiment, they were the only cell lines cultivated in our lab. The cells were cultured in Dulbecco’s modified Eagle medium, supplemented with 10% fetal calf serum, 50 µg/mL gentamicin, and 5 ng/mL amphotericin (all from Biochrom AG, Berlin, Germany), at 37 °C, in atmosphere of 5% CO_2_. The culture medium was replaced twice weekly.

Dose selection was informed by our previous Caco-2 and BJ monolayer study, which examined free Mel over 0–20 µg/mL and MNPs or MNPs-Mel over 0–400 µg/mL, including nanoparticle concentrations of 50, 100, 200, and 400 µg/mL [7]. The present spheroid experiments used free Mel at 2.5 µg/mL and MNPs or MNPs-Mel at a nominal nanoparticle concentration of 50 µg/mL. These concentrations were selected to produce measurable biochemical effects while retaining sufficient cellular structure for a detailed comparison of treatment-associated ultrastructural changes. This allowed the metabolic findings to be interpreted alongside organelle and cellular morphology in the same experimental model. The earlier concentration series provides a rationale for these exposures; dose–response relationships and an optimal dose in the tricellular spheroid model remain to be established. This dose was also in agreement with literature data reporting in vitro results (1–10 µg/mL) [28,42,73]. It was also calculated that the amount of 2.5 µg Mel was attached to 50 µg of MNPs, resulting thus in a dose of 50 µg MNPs-Mel/mL. And consequently, the dose of MNPs (without Mel) was also established to 50 µg/mL for a proper comparison of effects.

To test the ability of the three types of cells to internalize the MNPs-Mel, they were separately incubated with 50 µg/mL MNPs-Mel for 4 h. At the end of the incubation time, the cells were trypsinized for 5 min, centrifuged for 7 min at 500× *g*, and further processed for TEM.

A triple cellular tumor spheroid model was developed by using the three cell lines mentioned above and a scaffold of rat tail tendon collagen-I (Corning, Bedford, MA, USA). Briefly, cells were collected by trypsinization, counted and resuspended into fetal calf serum (from Gibco, Thermo Fisher Scientific, Waltham, MA, USA) at a ratio of Caco-2: BJ: HUVEC = 3:1:1, to make a final number of 1.2 × 10^6^ cells/spheroid in a volume of 50 µL. Then, cells were thoroughly mixed with the collagen-I gel (collagen-I: Hank’s balanced salt solution: fetal calf serum = 8:1:1), immediately placed into low-adherence, spherical-bottom 6-well plates (Nunclon sphera, Nunc, Thermo Fisher Scientific, Waltham, MA, USA) and allowed to polymerize for 30 min at 37 °C. Afterwards, medium was added and spheroids were accommodated for 24 h in standard conditions. Then, the plaques, containing the spheroids were put on an OrganoFlow plaque rocker (Mimetas, Oegstgeest, The Netherlands), to mimic flow conditions and to allow better nutrition of the spheroids for additional 9 days. Medium was changed every other day. After 10 days, the spheroids-containing matrices—simply called spheroids—which were used for experiments.

### 4.3. In Vitro Magnetic Hyperthermia

To explore the impact of the MH exposure on the Mel release from the MNPs-Mel, a spectral comparison was first performed, where a loading reference was calculated by subtracting the post-functionalization supernatant spectrum from that of the initial Mel solution, using the measurements described in Section 4.1. Next, two freshly prepared MNPs-Mel suspensions were evaluated at an MNP concentration of 0.5 mg/mL in cell-free medium without the three-dimensional matrix. This higher concentration was chosen to increase the amount of Mel available for a more precise spectroscopic assessment. The suspensions were exposed for 10 min to an AMF at frequency of 355 kHz and amplitude of 25 kA/m in an Easy Heat 0224 MH system (Ambrell, Scottsville, NY, USA), while temperature–time curves were recorded. After exposure, the nanoparticles were magnetically separated, and the recovered supernatants were examined by UV–Vis spectroscopy, focusing on the Mel-associated absorption region near 280 nm, in order to emphasize the Mel removed from the MNPs.

Untreated spheroids, and the spheroids incubated with 50 µg/mL MNPs for 24 h or 72 h, or with 50 µg/mL MNPs-Mel for 24 h or 72 h, were washed with a fresh culture medium, carefully transferred in 0.4 mL culture medium in 1.5 mL Eppendorf tubes and exposed for 30 min to an AFM using the settings specified above, in the same MH system (*n* = 3 matrices/group). Spheroids incubated without nanoparticles and subjected to the same AMF exposure conditions served as the AMF-only control group (Control-MH), allowing assessment of the response to field exposure in the absence of nanoparticles. Spheroids only exposed to medium were used as controls (*n* = 3). For each MH experiment, unexposed replicates were maintained in similar conditions, throughout the duration of the experiment. For the detailed procedure and selection of MH conditions, see [7]. Following MH exposure, spheroids were carefully put into fresh plates and further incubated in complete medium for an additional 24 h. After the 24 h or 72 h incubation with Mel, MNPs, and MNPs-Mel, following the 30 min AMF exposure and after an additional 24 h incubation post-MH exposure, the culture media were removed and stored at –80 °C for the ELISA molecular measurements.

Surface temperatures were recorded during the spheroid exposure protocol described above. We have plotted the heating curves during spheroid exposure to magnetic hyperthermia by measuring the temperature next to the spheroids containing matrices—compared to the control sample, which was maintained at 37.6 °C. The temperature was monitored using the fiber-optic temperature probe incorporated within the MH system, with a resolution of 0.1 °C. The probe was positioned in the middle of the colloidal nanoparticle suspension or, in the spheroid experiments, in proximity of the spheroids containing matrices—i.e., the probe did not penetrate the spheroids themselves.

### 4.4. Experimental Groups

The following experimental groups were taken into consideration. The first set of 3 groups contained cells from BJ, HUVEC and Caco-2 lines incubated for 4 h for testing MNPs-Mel internalization. The main spheroid experiment comprised five treatment groups (Mel, MNPs, MNPs-MH, MNPs-Mel and MNPs-Mel-MH) and two control groups: untreated Control and the AMF-only control (Control-MH). The seven experimental conditions were evaluated after initial incubation intervals of 24 and 72 h. Mel was used at 2.5 µg/mL, and MNPs and MNPs-Mel at 50 µg/mL. The groups designated MH received AMF exposure for 30 min at 355 kHz and 25 kA/m. Control-MH received the same AMF exposure without Mel or nanoparticles. The 24 h and 72 h labels refer to the initial incubation interval, not the duration of AMF exposure.

### 4.5. Viability Assay

Viability of cells in the spheroids from all experimental groups was evaluated by colorimetry with CellTiter 96^®^ AQueous Non-Radioactive Cell Proliferation Assay (Promega Corporation, Madison, WI, USA) and using a SpectraMax ID3 Multi-Mode Microplate Reader (Molecular Devices, San Jose, CA, USA) at 540 nm. Four replicates (sets of 4 spheroids/group) were performed for each experimental group, and data are presented as mean ± SD.

### 4.6. Toxicity Assay—ATP Measurement

The amount of ATP produced by spheroids from all experimental groups was measured using the CellTiter-Glo Luminescent Cell Viability Assay (Promega, Madison, WI, USA), as indicated by the producer, and with the SpectraMax ID3 Multi-Mode Microplate Reader (Molecular Devices, San Jose, CA, USA). Four replicates were performed for each experimental group, and data are presented as mean ± SD.

### 4.7. Angiogenesis Assay—VEGF and HIF-1α Measurements

The levels of the angiogenetic markers VEGF and HIF1 α from the supernatant (see Section 4.3) were measured by ELISA using the kits EH0327 Human VEGF (Vascular Endothelial Cell Growth Factor) ELISA Kit and EH0551 Human HIF1A (Hypoxia-inducible factor 1-alpha) ELISA Kit (Fine Test, Fine Biotech Co., Wuhan, China) as indicated by the manufacturer. Results are presented as mean ± SD from three independent experiments.

### 4.8. Apoptosis Induction—Caspase-9 Measurement

The levels of caspase-9,involved in the mitochondrial apoptotic pathway were measured with caspase 9 (EH0650) ELISA Immunoassay kit (Fine Test, Fine Biotech Co., Wuhan, China) as indicated by the manufacturer. Data are presented as mean ± SD. Three independent replicates were performed for each experimental group.

### 4.9. Statistical Analysis

Raw MTS absorbance at 540 nm, ATP-associated luminescence values and ELISA values were analyzed using ordinary two-way ANOVA. The analysis included four separately processed spheroid containing matrices/well measurements per treatment-time cell for MTS and ATP (*n* = 4, independent biological experiments) and three separately processed measurements per cell for VEGF, HIF-1α, and caspase-9 (*n* = 3, independent biological experiments). Results are reported as raw means with Tukey-adjusted *p* values; statistical significance was defined as *p* < 0.05.

The statistical factor experimental condition comprised seven levels: Control, Control-MH, Mel, MNPs, MNPs-Mel, MNPs-MH and MNPs-Mel-MH. Initial incubation interval (24 or 72 h) was the second factor. Reporting was restricted to the following contrasts of interest: each treatment vs. control; MNPs vs. MNPs-MH; MNPs-Mel vs. Mel; MNPs-Mel vs. MNPs; and MNPs-Mel vs. MNPs-Mel-MH. The current analyses used ordinary two-way ANOVA with the condition × interval interaction. MTS and ATP were analyzed as percentages of the untreated 24 h control reference, whereas VEGF, HIF-1α and caspase-9 were analyzed as concentrations. Statistical analyses were performed using GraphPad Prism version 11.0.2 (Insight Partner, New York, NY, USA). Tukey-adjusted comparisons among all seven conditions were performed separately within each interval, giving two families of 21 pairwise comparisons per endpoint. All reported pairwise *p* values were adjusted; significance was defined as *p* < 0.05.

### 4.10. Nanoparticle Internalization and Ultrastructural Evaluation of Caco-2 Cells in Spheroids

After the 5 min trypsinization consecutive to the 4 h incubation with MNPs-Mel, the cells in the three lines were suspended in 1.5% osmium tetroxide (Sigma-Aldrich, St. Louis, MO, USA) in 0.15 M phosphate buffer (pH 7.4) for a 1.5 h fixation. The cellular suspensions were centrifuged (7 min at 500× *g*), the supernatant was completely removed, and the pellets were further processed. At the end of the various incubation intervals (with or without MH), 2 matrices with tens of spheroids each per group were further processed for TEM, with analysis of 2 spheroids from each matrix. They were prefixed for 2 h with 2.7% glutaraldehyde (Agar Scientific Ltd., Stansted, UK) in 0.1 M phosphate buffer (pH 7.4), washed four times with 0.1 M phosphate buffer (3 × 1 h and 1 × 24 h), and postfixed for 1.5 h with 1% osmium tetroxide in 0.15 M phosphate buffer. Next, all the samples (cell pellets and spheroids) were dehydrated in acetone series (50–100%, 30 min each), infiltrated with acetone solutions (30%, 50%, 75% 1 h each and pure resin overnight) of EMBed-812 (Electron Microscopy Sciences, Hatfield, PA, USA). The polymerized blocks were cut with an Ultra 45° diamond knife (DiATOME AG, Nidau, Switzerland) on a Bromma 8800 ULTRATOME III ultramicrotome (LKB Produckter AB, Stockholm-Bromma, Sweden). To demonstrate the actual spheric shape of the morphology and general structure of the obtained spheroids, from a random spheroid in the control group, semithin sections were cut at 200 nm, placed on glass slides, stained with 1% methylene blue and examined with a Nikon Eclipse 80i light microscope (Nikon Corporation, Tokyo, Japan). Forty different images were photographed at two magnifications (objectives × 40 and × 100) with an Olympus Color View 1 CCD camera (Olympus Soft Imaging Solutions GMBH, Münster, Germany) running with CellD Olympus software, version 3.1 (Olympus Soft Imaging Solutions GMBH, Münster, Germany). Ultrathin sections were subsequently cut from all the samples (cells and spheroids) at 60–70 nm and collected on 300 mesh Ø3 mm copper grids (Agar Scientific Ltd., Stansted, UK). After double contrasting for 10 min with 13% uranyl acetate (Merck, Darmstadt, Germany) and for 5 min with 2.8% lead citrate (Fluka AG, Buchs, Switzerland), 50–70 sections were examined per each matrix, with the same transmission electron microscope mentioned in Section 4.1.

## 5. Conclusions

Melittin-functionalized Fe_3_O_4_ nanoparticles combined with AMF exposure produced a pronounced biochemical and ultrastructural response in collagen I-based Caco-2/BJ/HUVEC spheroids. The combined treatment yielded the lowest ATP signal and VEGF concentration at both intervals and the lowest MTS signal at 72 h. Its higher MTS signal relative to MNPs-Mel alone at 24 h indicates that the early response differed between metabolic endpoints. The caspase-9 increase and the transient HIF-1α response further show that the soluble-marker profile evolved with the incubation interval. Marked mitochondrial cristae damage, matrix rarefaction and cellular disruption provide structural context for the metabolic findings. Together, the results support substantial cellular injury with mitochondrial involvement in the combined-treatment group. The biochemical measurements represent the integrated response of the tricellular spheroid, and the specific molecular cell-death pathway remains an interpretation of these findings rather than a directly established mechanism.

## Data Availability

The original contributions presented in this study are included in the article/Supplementary Materials. Further inquiries can be directed to the corresponding author.

## Supplementary Materials

The following supporting information can be downloaded at https://www.mdpi.com/article/10.3390/ijms27209302/s1.

## Abbreviations

The following abbreviations are used in this manuscript:

Control-MH 24 h	Control spheroids exposed to AMF 30 min at 25 kA/m for 24 h
Control-MH 72 h	Control spheroids exposed to AMF 30 min at 25 kA/m for 72 h
BJ cells	Human dermal fibroblasts
Caco-2 cells	Caucasian colon adenocarcinoma cells
HIF-1α	Hypoxia-inducible factor 1 alpha
HUVEC	Human umbilical vein endothelial cell
Mel	Melittin
Mel 24 h	Spheroid group incubated for 24 h with 2.5 µg/mL Mel
Mel 72 h	Spheroid group incubated for 72 h with 2.5 µg/mL Mel
MH	Magnetic hyperthermia
MNPs	Magnetic nanoparticles
MNPs 24 h	Spheroid group incubated for 24 h with 50 µg/mL MNPs
MNPs 72 h	Spheroid group incubated for 72 h with 50 µg/mL MNPs
MNPs-MH 24 h	Spheroid group incubated for 24 h with 50 µg/mL MNPs exposed to MH 30 min at 25 kA/m
MNPs-MH 72 h	Spheroid group incubated for 72 h with 50 µg/mL MNPs exposed to MH 30 min at 25 kA/m
MNPs-Mel	Melittin-functionalized MNPs
MNPs-Mel 24 h	Spheroid group incubated for 24 h with 50 µg/mL MNPs-Mel
MNPs-Mel 72 h	Spheroid group incubated for 72 h with 50 µg/mL MNPs-Mel
MNPs-Mel-MH 24 h	Spheroid group incubated for 24 h with 50 µg/mL MNPs-Mel exposed to MH 30 min at 25 kA/m
MNPs-Mel-MH 72 h	Spheroid group incubated for 72 h with 50 µg/mL MNPs-Mel exposed to MH 30 min at 25 kA/m
VEGF	Vascular endothelial growth factor
TEM	Transmission electron microscopy
