# Supplementary material for: Antitumor Effects of Melittin-Functionalized Fe3O4 Magnetic Nanoparticles in Collagen-I-Based Caco-2/BJ/HUVEC Spheroids Enhanced by Magnetic Hyperthermia
Source: Int J Mol Sci. 2026 Oct 20;27(20):9302. doi: 10.3390/ijms27209302 (PMC13705337; doi:10.3390/ijms27209302)
Supplement: Supplementary file 1 [file ijms-27-09302-s001.zip › ijms-4575968-supplementary.pdf]

International Journal of *Molecular Sciences*

MDPI

Supplementary Materials

Antitumor Effects of Melittin-Functionalized Fe_3O_4 Magnetic Nanoparticles in Collagen-I-Based Caco-2/BJ/HUVEC Spheroids Enhanced by Magnetic Hyperthermia

Alex Câmpian [1], Ioana Bâldea [2,*], Mara Muntean [1], Cristian Iacoviță [3], Nicoleta Decea [2] and Adrian Florea [1]

1. Department of Cell and Molecular Biology, Faculty of Medicine, "Iuliu Hațieganu" University of Medicine and Pharmacy, 6 Louis Pasteur St., 400349 Cluj-Napoca, Romania; alexcampian@yahoo.com (A.C.); muntean.mara@elearn.umfcluj.ro (M.M.); aflorea@umfcluj.ro (A.F.)
2. Department of Physiology, Faculty of Medicine, "Iuliu Hațieganu" University of Medicine and Pharmacy, 1–3 Clinicilor St., 400012 Cluj-Napoca, Romania; nicoleta.decea@yahoo.com
3. Department of Pharmaceutical Physics-Biophysics, Faculty of Pharmacy, "Iuliu Hațieganu" University of Medicine and Pharmacy, 6 Louis Pasteur St., 400349 Cluj-Napoca, Romania; cristian.iacovita@umfcluj.ro

* Correspondence: ioana.baldea@umfcluj.ro

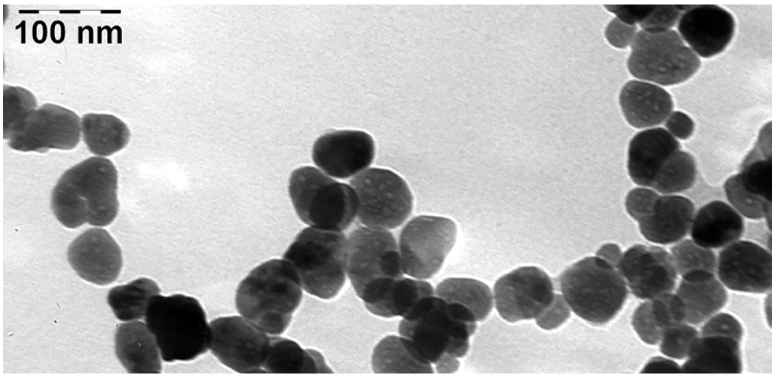

Figure S1. Representative TEM image of MNPs-Mel.

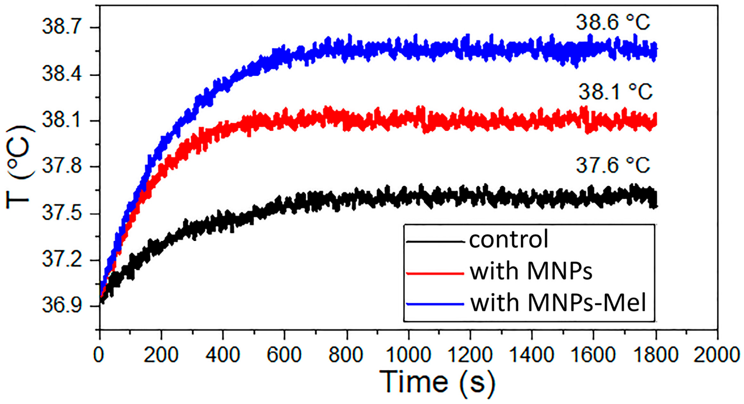

Figure S2. Heating curves during spheroids exposure to MH. The temperature was monitored using the fiber-optic temperature probe incorporated within the MH system, with a resolution of 0.1 °C. The probe was positioned in proximity to the spheroids containing matrices (n = 3).

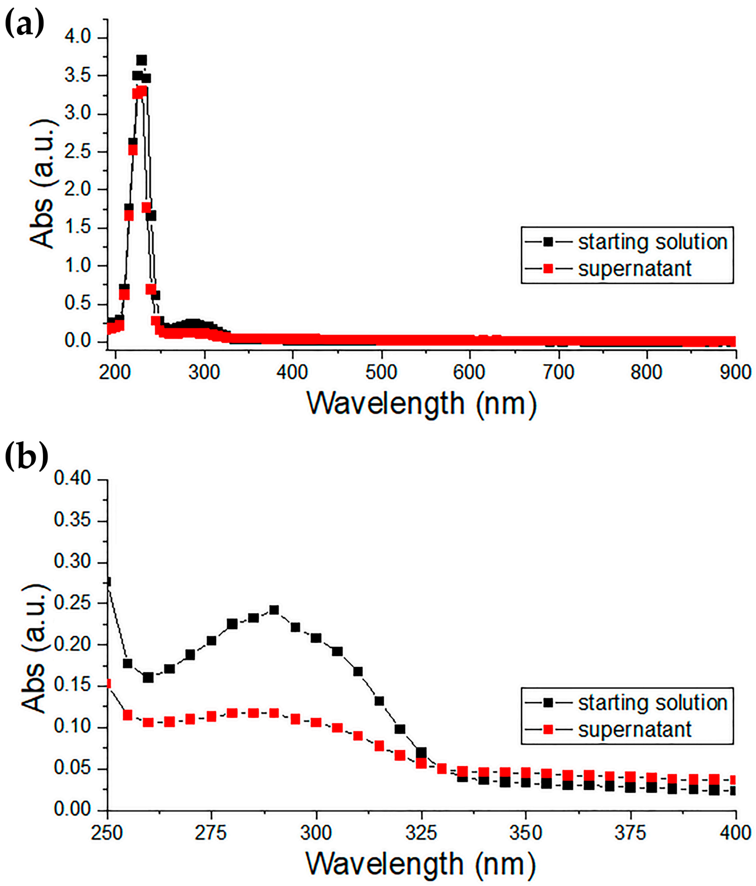

Figure S3. UV-Vis spectra of starting solution and supernatant upon attachment Mel procedure: Raw spectra (a) and Zoom-in UV-Vis spectra to highlight the peak at 280 nm (b).

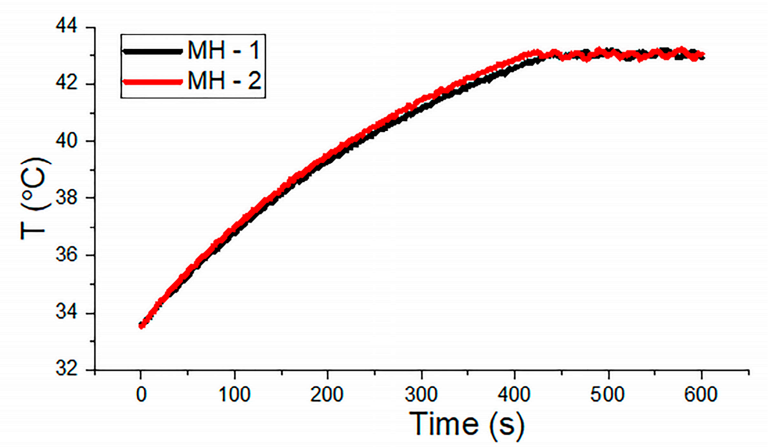

Figure S4. Heating curves of two samples of MNPs-Mel (0.5 mg/mL) during MH exposure (25kA/m, 355 kHz).

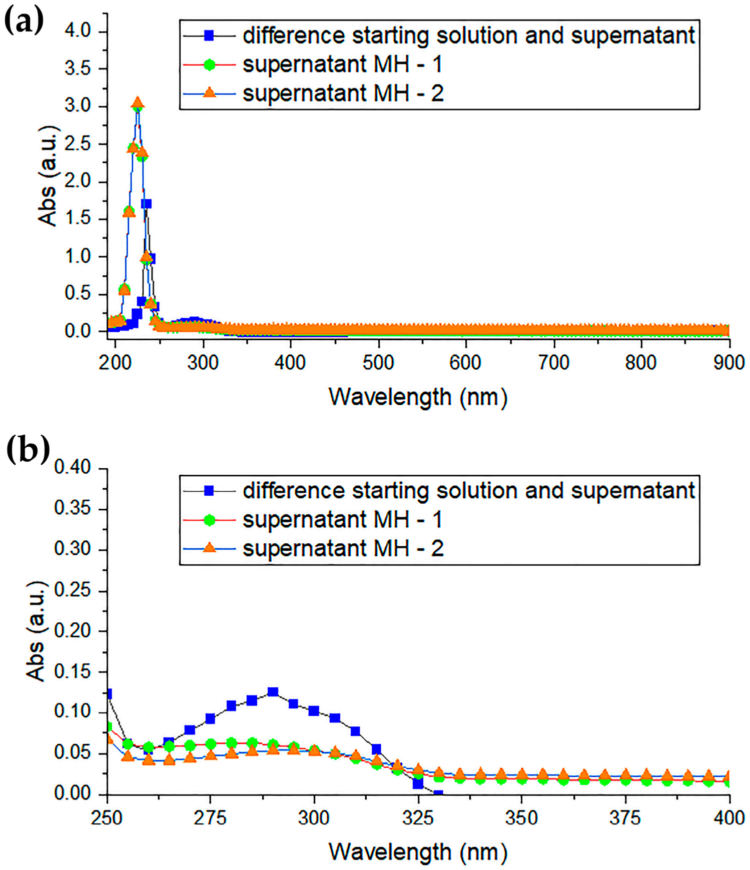

Figure S5. UV-Vis spectra as difference between starting solution and supernatant upon attachment Mel procedure and supernatant solutions upon MH exposure. Raw spectra (a) and Zoom-in UV-Vis spectra to highlight the peak at 280 nm (b).

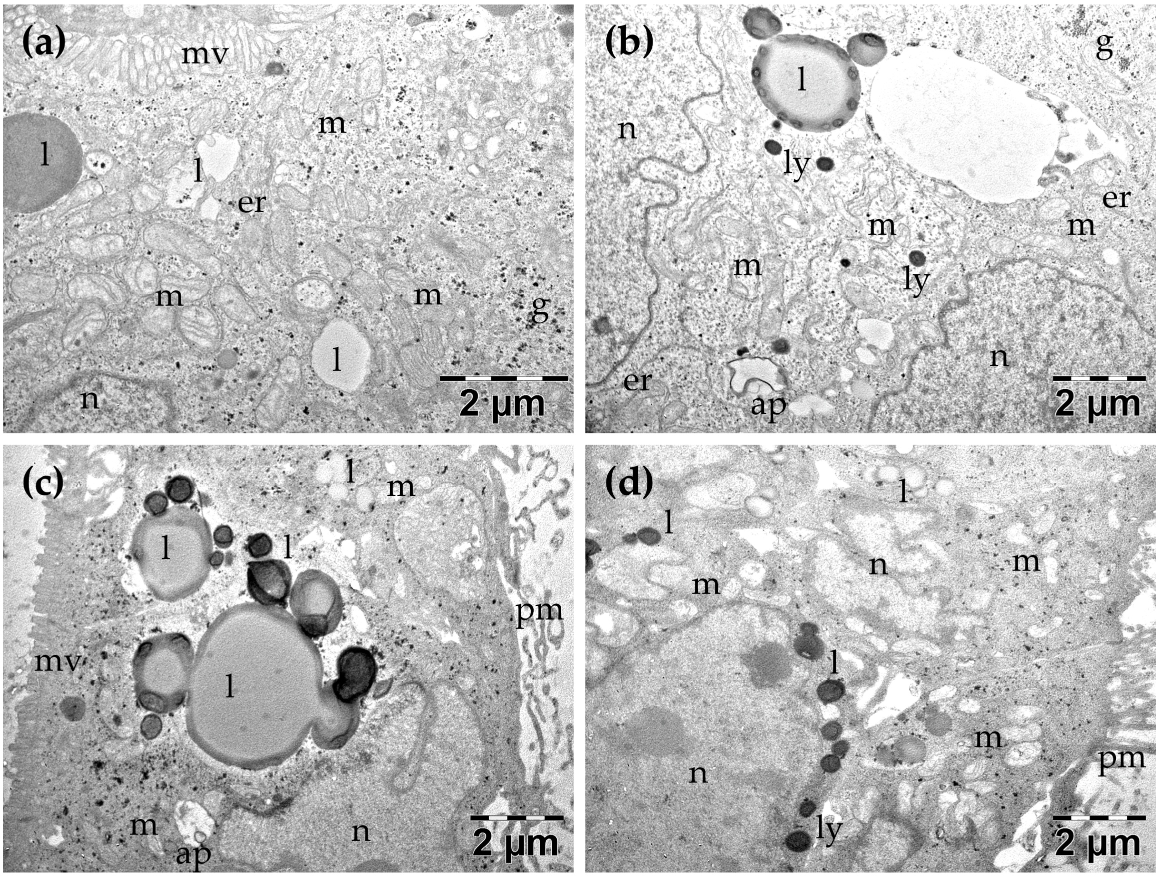

Figure S6. Representative TEM images of cells in spheroids from control groups exposed to AMF for 24 h (a,b) or 72 h (c,d). ap: autophagosome; er: endoplasmic reticulum; g: glycogen; l: lipid droplets; ly: lysosomes; m: mitochondria; mv: microvilli; n: nucleus; pm: plasma membrane.

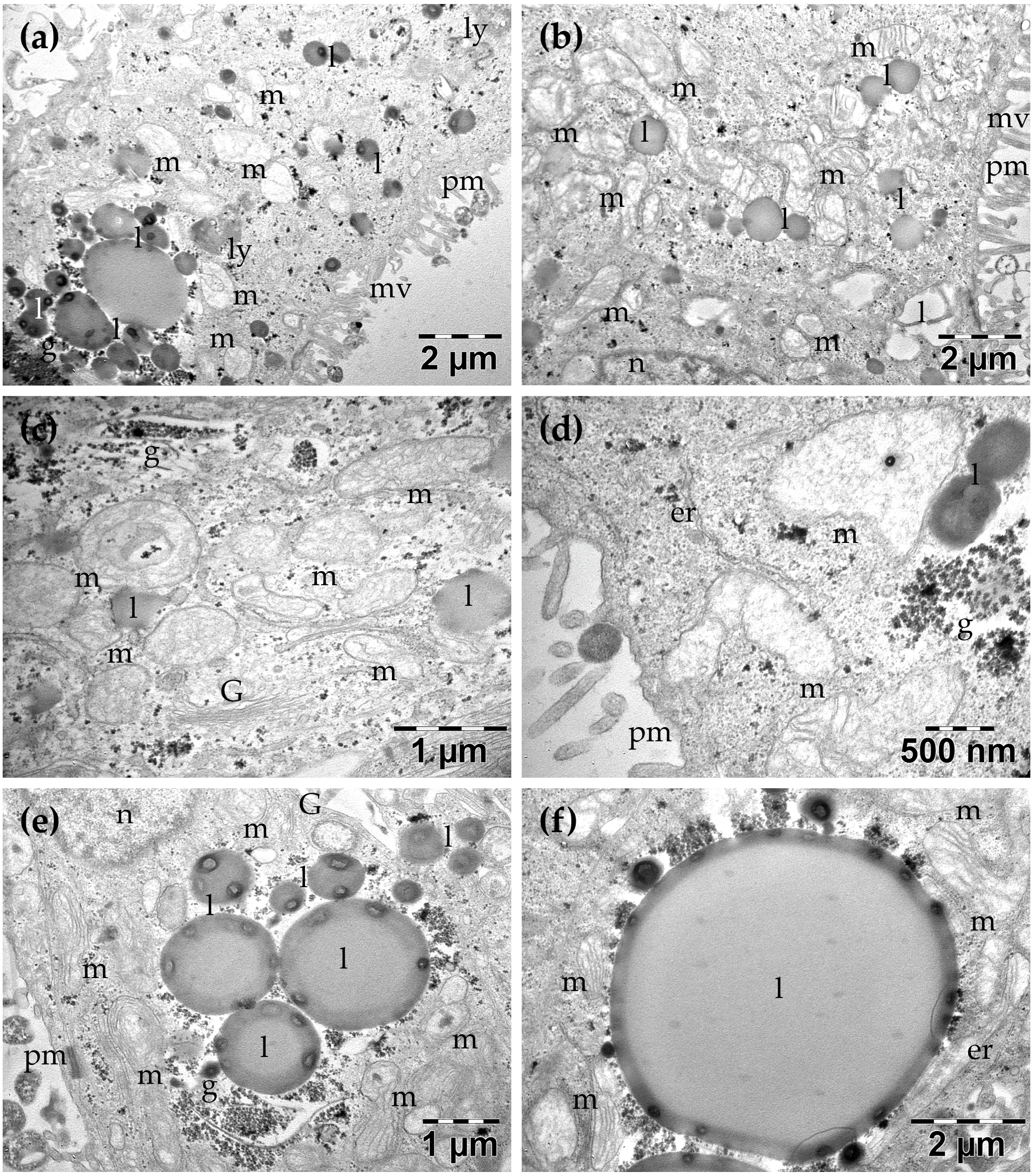

Figure S7. (a–f) Representative TEM images of cells in spheroids from Mel 24 h group. er: endoplasmic reticulum; G: Golgi apparatus; g: glycogen; l: lipid droplets; ly: lysosome; m: mitochondria; mv: microvilli; n: nucleus; pm: plasma membrane.

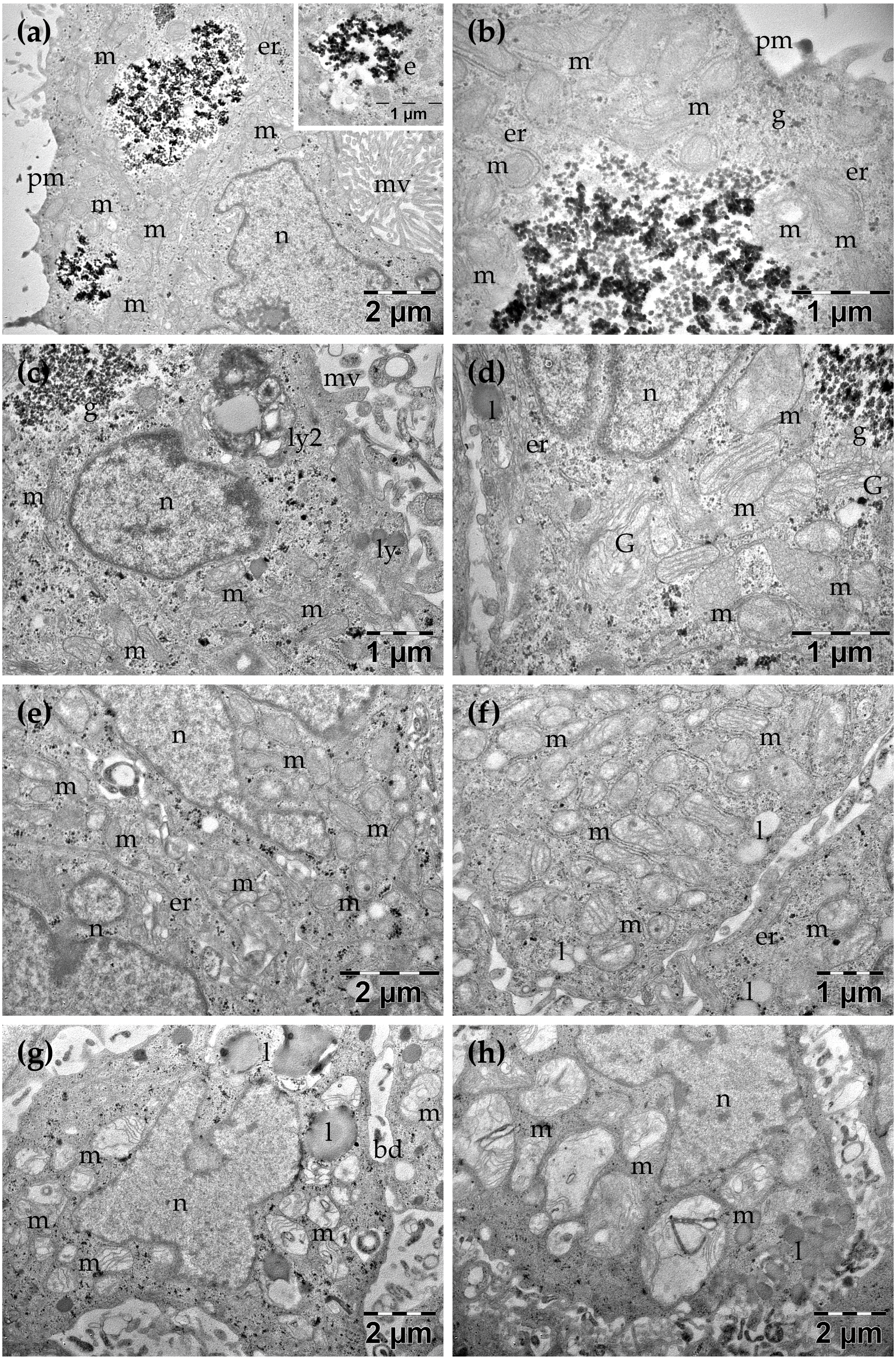

Figure S8. Representative TEM images of cells in spheroids from MNPs 24 h (a,b), MNPs-MH 24 h (c,d), MNPs 72 h (e,f) and MNPs-MH 72 h (g,h) groups. e: endosome; er: endoplasmic reticulum; G: Golgi apparatus; g: glycogen; l: lipid droplets; ly: lysosome; ly2: secondary lysosome; m: mitochondria; mv: microvilli; n: nucleus; pm: plasma membrane.

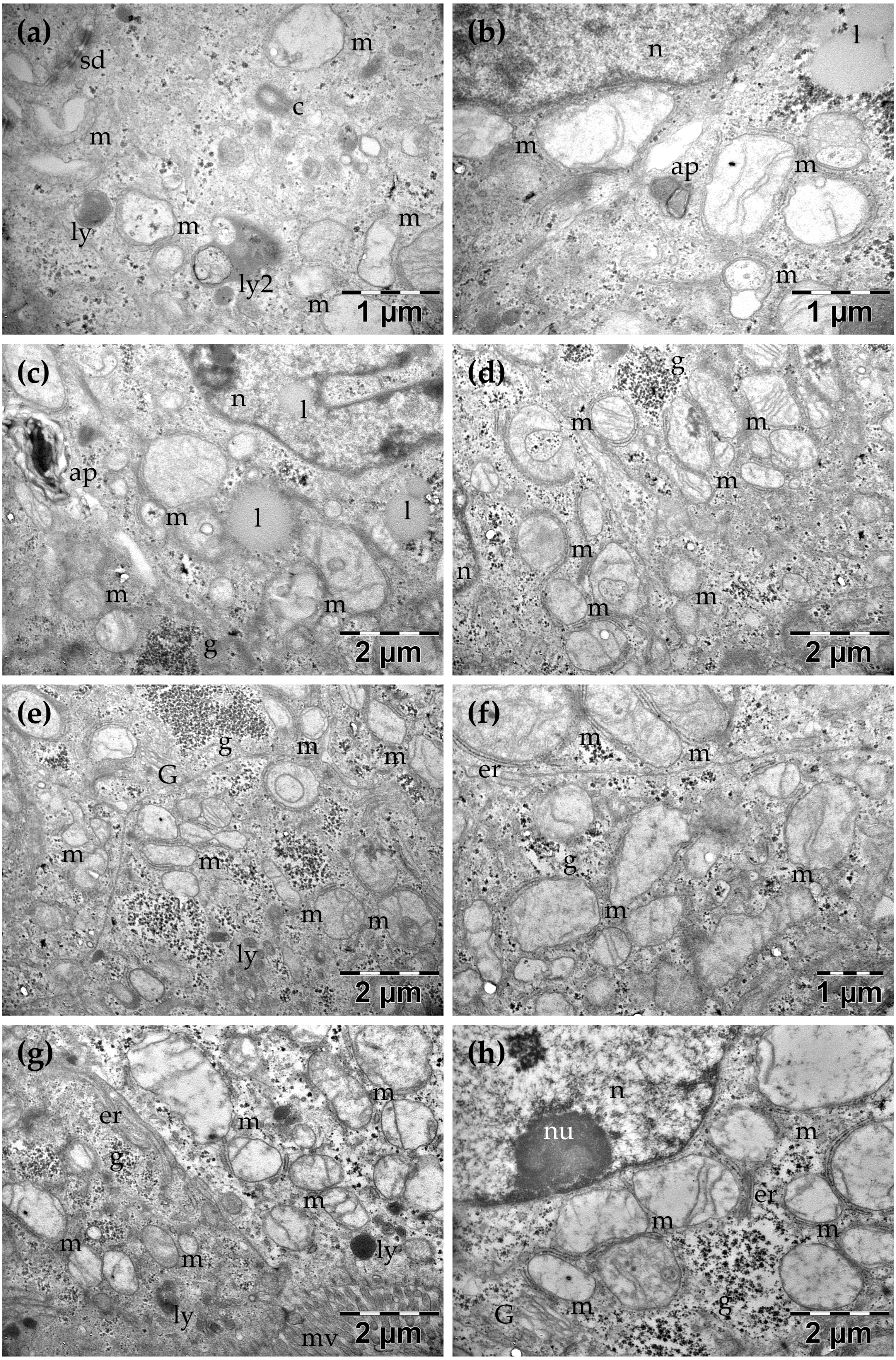

Figure S9. (a–h) Representative TEM images of cells in spheroids from MNPs-Mel 24 h group. ap: autophagosome; c: centriole; er: endoplasmic reticulum; G: Golgi apparatus; g: glycogen; l: lipid droplets; ly: lysosome; m: mitochondria; mv: microvilli; n: nucleus; nu: nucleolus; sd: spot desmosomes.

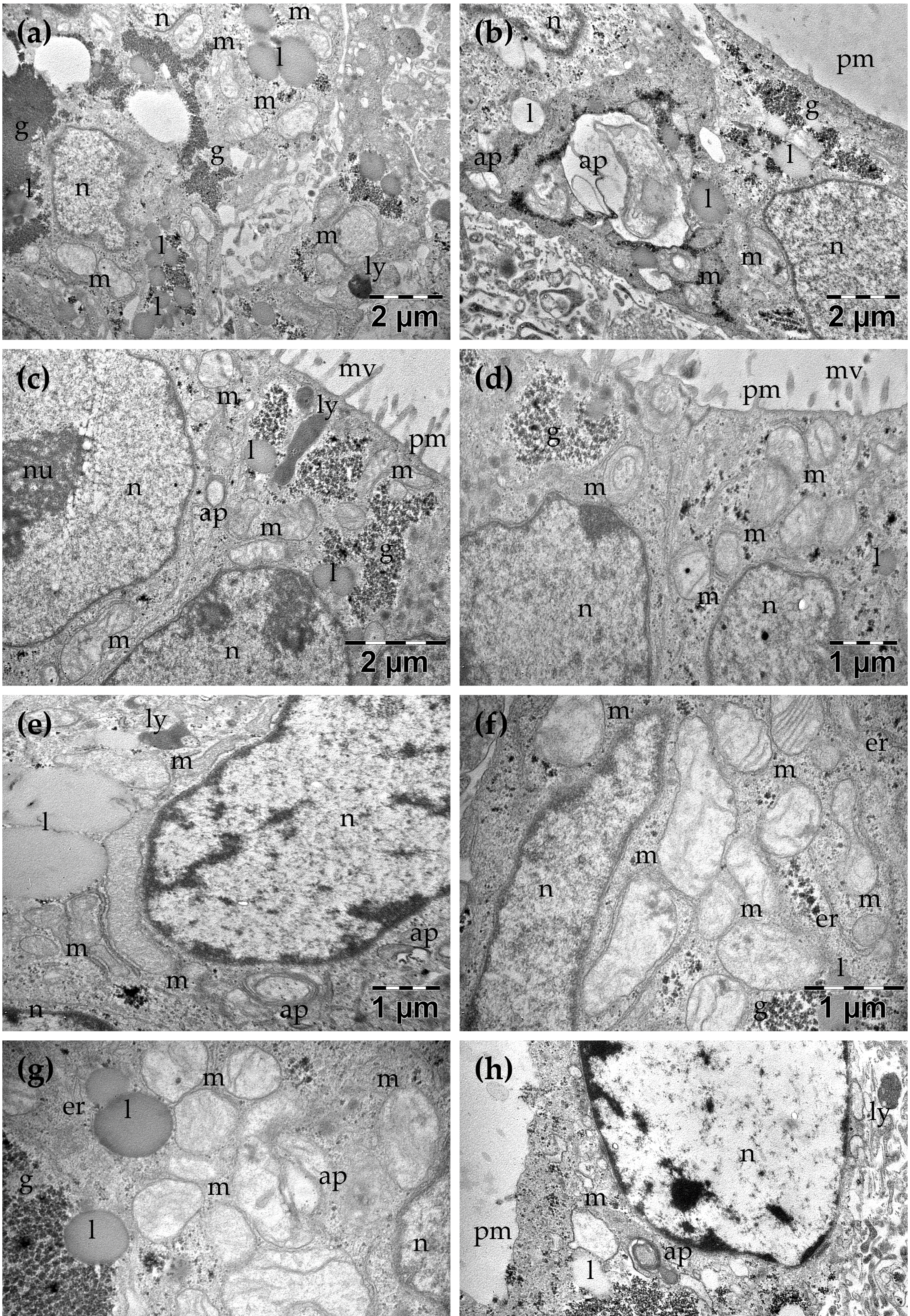

Figure S10. (a–h) Representative TEM images of cells in spheroids from MNPs-Mel-MH 24 h group. ap: autophagosome; er: endoplasmic reticulum; g: glycogen; l: lipid droplets; ly: lysosome; m: mitochondria; mv: microvilli; n: nucleus; nu: nucleolus; pm: plasma membrane.
